# Initiatives, Concepts, and Implementation Practices of the Findable, Accessible, Interoperable, and Reusable Data Principles in Health Data Stewardship: Scoping Review

**DOI:** 10.2196/45013

**Published:** 2023-08-28

**Authors:** Esther Thea Inau, Jean Sack, Dagmar Waltemath, Atinkut Alamirrew Zeleke

**Affiliations:** 1 Department of Medical Informatics Institute for Community Medicine University Medicine Greifswald Greifswald Germany; 2 International Health Department Johns Hopkins Bloomberg School of Public Health Baltimore, MD United States

**Keywords:** data stewardship, findable, accessible, interoperable, and reusable data principles, FAIR data principles, health research, Preferred Reporting Items for Systematic Reviews and Meta-Analyses, PRISMA, qualitative analysis, scoping review, information retrieval, health information exchange

## Abstract

**Background:**

Thorough data stewardship is a key enabler of comprehensive health research. Processes such as data collection, storage, access, sharing, and analytics require researchers to follow elaborate data management strategies properly and consistently. Studies have shown that findable, accessible, interoperable, and reusable (FAIR) data leads to improved data sharing in different scientific domains.

**Objective:**

This scoping review identifies and discusses concepts, approaches, implementation experiences, and lessons learned in FAIR initiatives in health research data.

**Methods:**

The Arksey and O’Malley stage-based methodological framework for scoping reviews was applied. PubMed, Web of Science, and Google Scholar were searched to access relevant publications. Articles written in English, published between 2014 and 2020, and addressing FAIR concepts or practices in the health domain were included. The 3 data sources were deduplicated using a reference management software. In total, 2 independent authors reviewed the eligibility of each article based on defined inclusion and exclusion criteria. A charting tool was used to extract information from the full-text papers. The results were reported using the PRISMA-ScR (Preferred Reporting Items for Systematic Reviews and Meta-Analyses extension for Scoping Reviews) guidelines.

**Results:**

A total of 2.18% (34/1561) of the screened articles were included in the final review. The authors reported FAIRification approaches, which include interpolation, inclusion of comprehensive data dictionaries, repository design, semantic interoperability, ontologies, data quality, linked data, and requirement gathering for FAIRification tools. Challenges and mitigation strategies associated with FAIRification, such as high setup costs, data politics, technical and administrative issues, privacy concerns, and difficulties encountered in sharing health data despite its sensitive nature were also reported. We found various workflows, tools, and infrastructures designed by different groups worldwide to facilitate the FAIRification of health research data. We also uncovered a wide range of problems and questions that researchers are trying to address by using the different workflows, tools, and infrastructures. Although the concept of FAIR data stewardship in the health research domain is relatively new, almost all continents have been reached by at least one network trying to achieve health data FAIRness. Documented outcomes of FAIRification efforts include peer-reviewed publications, improved data sharing, facilitated data reuse, return on investment, and new treatments. Successful FAIRification of data has informed the management and prognosis of various diseases such as cancer, cardiovascular diseases, and neurological diseases. Efforts to FAIRify data on a wider variety of diseases have been ongoing since the COVID-19 pandemic.

**Conclusions:**

This work summarises projects, tools, and workflows for the FAIRification of health research data. The comprehensive review shows that implementing the FAIR concept in health data stewardship carries the promise of improved research data management and transparency in the era of big data and open research publishing.

**International Registered Report Identifier (IRRID):**

RR2-10.2196/22505

## Introduction

### Background

The vast amount of data obtained from research would benefit the larger scientific community more if it were easily findable, accessible, interoperable, and reusable (FAIR) [1]. However, most research data are still maintained in individualized silos across the health care continuum instead of being managed in interoperable and integrated knowledge bases [2]. The COVID-19 global crisis partially improved data sharing to support the secondary and integral use of the available data across the globe and disciplines [3,4]. Secondary data reuse has been shown to be a key enabler for more extensive and valuable research dimensions, especially in situations where the data are scarce, sparse, heterogeneous, and sensitive with regard to privacy [5,6].

### Objectives

In this study, we conducted a scoping review to analyze the approaches used to FAIRify health research data, the various software used, the challenges faced and the mitigation strategies used to navigate these challenges, and the networks actively involved. The results of this work will provide valuable insights for stakeholders to reference when seeking to influence organizational practices that promote FAIR practices, FAIRify health research data, develop related software, seek collaborations with networks actively involved in the implementation of FAIR data principles, or evaluate the FAIRness of a particular set of health research data.

## Methods

This scoping review adopted the framework outlined by Arksey and O’Malley [7]. It includes the following steps: (1) identifying the research question; (2) identifying relevant studies; (3) study selection; (4) charting the collected data; and (5) collating, summarizing, and reporting the results.

### Stage 1: Identifying the Research Questions

Our pilot literature exploration included published works in PubMed, Google Scholar, and Web of Science. We used FAIR data principle keywords to match Medical Subject Headings (MeSH) used to tag PubMed peer-reviewed literature, along with combinations of terms used in clinical research, public health, health care, pharmacology, and patient data. The bibliographies of key papers were scrutinized for other complementary publications, and recurrent alerts for this exploration were set up on the 3 databases.

Our informal desk review showed that there is indeed a growing interest in following the phases of the research life cycle [8,9]. These findings motivated us to better understand the approaches used in the implementation of the FAIR data principles and their impact on the way research in health will be conducted, subsequently leading to our research questions. We decided that the review should only include studies that showed either an actual approach to implement the FAIR data principles in the health domain or the recorded results of the implementation of the FAIR data principles. The review excluded studies that introduced or provided an overview of the FAIR principles. Studies that showed the implementation of the principles in a domain other than health were also excluded.

The general objective of this work was to conduct a scoping review to identify concepts, approaches, implementation experience, and lessons learned from the FAIR data principle initiatives in the health domain. The following research questions were formulated to meet the objectives:

1. What approaches are being used or piloted in the implementation of the FAIR data principles in the health data domain since the conception of these principles in 2014?
2. What are the challenges and risks with regard to the approaches used in the practical implementation of the FAIR data principles in the health data domain?
3. What are the suggested concepts and approaches to mitigate concerns regarding the implementation of the FAIR data principles in the health data domain?
4. Which are the active public and private research and service networks involved in the implementation of the FAIR data principles in the health data domain?
5. What are the reported outcomes in terms of data sharing, data reuse, and research publication after the implementation of the FAIR data principles in the health data domain?

### Stage 2: Identifying Relevant Studies

With the aid of an experienced research librarian (JS), we identified relevant studies from 3 primary electronic databases: PubMed, Web of Science, and Google Scholar. The keywords for the scoping review search strategies were categorized into terms related to the FAIR data principles, data sharing, and health. Open terms were used for the construction of the search strategy for this study. The Boolean operators “AND” and “OR” were used to guide the search strategy. The following descriptors, keywords, and their combinations were used to construct the strategies: “health*,” “pharma*,” “research and development” (MeSH term), “research” (MeSH term), “biomedical research” (MeSH term), “data collection” (MeSH term), “metadata” (MeSH term), “registr*” (MeSH term), “registr*,” “Open access publishing,” “data curation,” “data preservation,” “data provenance,” “data*,” “data sharing,” “open science,” “repositor*,” “data management” (MeSH term), “FAIR data Principles” (title or abstract), “FAIR Principles,” “FAIR guiding Principles,” “Data stewardship,” and “Data management systems.” The search strategy we formulated for this purpose can be found in Multimedia Appendix 1.

The PRISMA-ScR (Preferred Reporting Items for Systematic Reviews and Meta-Analyses extension for Scoping Reviews) was used to report the findings [10]. The operational definition of “health” for this scoping review is based on the 2018 European Union (EU) General Data Protection Regulation (GDPR) and the health ecosystem components framed by the World Health Organization [11,12]. Accordingly, *health data* in this review is defined in the context of data from service and research practice in health services (clinical records, electronic health records and electronic medical records, prescribing, diagnostics, laboratory, health insurance, disease surveillance, immunization records, public health reporting, vital statistics, registries, clinical trials, clinical research, and public health research) [13].

As inclusion criteria, we considered literature published between January 1, 2014, and December 31, 2020. The start year of 2014 was chosen as FAIR concept initiatives and official publications first became available in that year. Moreover, to be included as a potential paper, the literature must be published in English and within the scope of FAIR principle application in the health domain (defined by the operational definition). Deduplication was performed by exporting all search results from web-based databases and gray literature sources to a reference management software. Unique search results were exported to a screening tool to facilitate an independent screening process.

### Stage 3: Study Selection

The Rayyan software (Rayyan Systems, Inc) was chosen as the primary screening tool to expedite the initial screening of abstracts and titles using a semiautomated process while incorporating a high level of usability [14]. Initial screening based on the inclusion criteria can be cumbersome. Rayyan provides a platform for the collaborative screening of publication abstracts and titles [14]. According to the inclusion and exclusion criteria, nonrelevant studies were excluded from the review at this point. Where the relevance of the publication was unclear from the title or abstract, the reviewer read the full publication to determine its eligibility. Further changes to the search criteria to improve the search findings were made at this stage as necessary. The eligible publications screened in the first stage were then independently read in full by 2 researchers to further determine the relevance of the publication content to the research questions. When an agreement could not be reached during the initial and full-text screening stages, an independent researcher was consulted. This was the basis on which a PRISMA (Preferred Reporting Items for Systematic Reviews and Meta-Analyses) flow diagram was then generated [10].

### Stage 4: Data Charting

A pretested data charting form shown in the protocol published before this review was used by the reviewers to determine which variables to extract [13]. This form provided flexibility for iterative updates during the data charting process. The “descriptive-analytical” approach, as described by Arksey and O’Malley [7], was used in the data collection process. In this process, the researchers critically examined the identified articles and documents that met the eligibility criteria and extracted the relevant data from each publication using the pretested charting form. The data were organized into a chart with 2 main sections. In the first section (Overview) we categorized the metadata of the included publications. In the second section (Research Questions) we extracted and included data based on our predetermined objectives [13].

### Stage 5: Collating, Summarizing, and Reporting the Results

This scoping review was focused on the range of data identified and curated. Quantitative assessment was limited to a count of the number of sources reporting a particular FAIR thematic issue or recommendation. After charting the relevant data from the studies on spreadsheets, the results were collated and described using summary statistics, charts, and figures. We also mapped the themes derived from the research questions (eg, FAIR implementation approaches, available FAIR networks, and FAIR infrastructural and security challenges) and other emerging themes during charting and analysis. Our results and implications for future research, practice, and policy were discussed accordingly.

### Ethical Considerations

No ethics approval was required for this work as only secondary data from published sources were included in the scoping review. The public was not invited to participate in any stage of this work.

## Results

### The PRISMA Chart

Our first search resulted in 1561 records. The deduplication process led us to eliminate 5.25% (82/1561) of the records. We read through the titles and abstracts with the help of Rayyan software and eliminated 89.94% (1404/1561) of the records.

**Figure 1:**
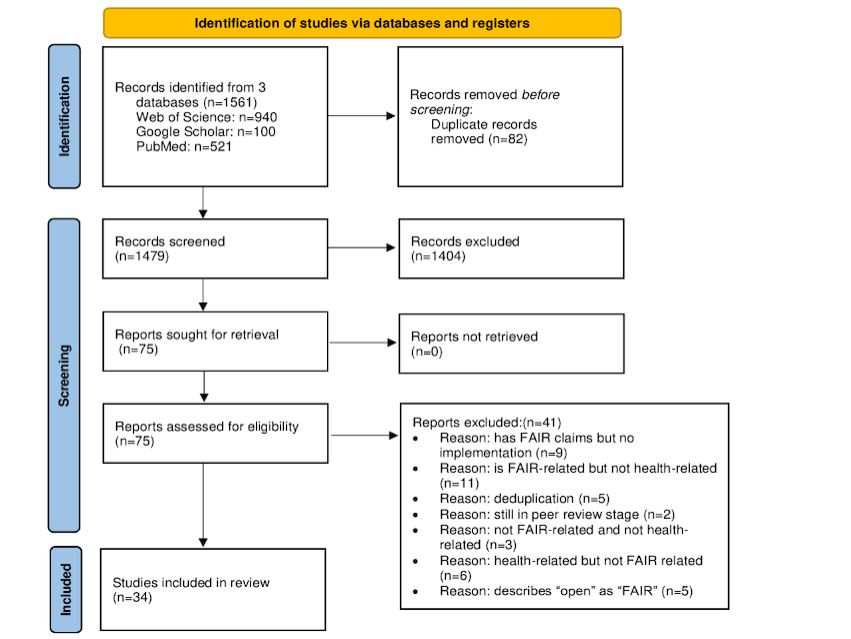
PRISMA (Preferred Reporting Items for Systematic Reviews and Meta-Analyses) chart showing the process of obtaining the relevant literature. FAIR: findable, accessible, interoperable, and reusable.

We then analyzed the full texts of the remaining 75 records and eliminated 41 (55%) based on relevance to the FAIR data principles in the health domain, as shown in Figure 1*.*

### General Observations

We included the year of publication, domain focus, countries where the work was conducted, and type of method that was used to conduct research or surveillance. We considered only the first author where there were multiple authors involved in the publication. On this basis, the United States topped the list with up to 38% (13/34) of publications [15-27]. Both Germany [28-32] and the Netherlands [33-37] had 15% (5/34) of related publications. France had 12% (4/34) of related publications [38-41]. We found only 3% (1/34) of related publications from Austria [42], Belgium [43], Greece [44], Portugal [45], Turkey [6], Uganda [46], and the United Kingdom [47]. We also found that most of the work on FAIR efforts in the health research domain was conducted in 2020, as shown in Table 1.

On the basis of the Systematized Nomenclature of Medicine–Clinical Terms (SNOMED-CT), a comprehensive, multilingual clinical health care terminology [48], we classified the research areas dealing with FAIR principles (Table 2). We listed the parents for medical specialties. However, we were not able to successfully map themes related to population health [15], demography [25], and general health data research [6,30,36] as we found SNOMED-CT to be lacking in these areas. Similarly, pharmacy [23] and pharmacovigilance [44] were not listed as medical specialties. We also found that biomedical themes were not exhausted in SNOMED-CT.

Regarding the study types, all the publications we reviewed were qualitative in nature except for the studies by van Panhuis et al [15] and Looten and Simon [39], which were mixed. In addition, all the publications we reviewed described work in which the FAIR principles had already been implemented, except for the study by Mons [35], which is a conceptual work.

**Table 1:** Number of easily findable, accessible, interoperable, and reusable (FAIR)–related publications in the health domain included after all the screening stages (2014-2020).

Year	Publications, n (%)
2014	0 (0)
2015	0 (0)
2016	1 (3)
2017	2 (6)
2018	10 (29)
2019	5 (15)
2020	16 (47)

**Table 2:** Classification of health domains based on the Systematized Nomenclature of Medicine–Clinical Terms (SNOMED-CT).

Parent and concept IDs			Children		Study
**Medical specialty (qualifier value); SCTID^a^: 394733009**					
	Cardiology (qualifier value); SCTID: 394579002	N/A^b^		Kass-Hout et al [19]	
	Medical oncology (qualifier value); SCTID: 394593009	N/A		Lacey et al [24] Kalendralis et al [37]	
	Radiation oncology (qualifier value); SCTID: 419815003	N/A		Traverso et al [34]	
	Emergency medicine (qualifier value); SCTID: 773568002	N/A		Bhatia et al [27]	
	Public health and preventive medicine (qualifier value); SCTID: 26081000087101	N/A		Mons [35] Zondergeld et al [36]	
	Infectious diseases (specialty; qualifier value); SCTID: 394807007	Tuberculosis (disorder); SCTID: 56717001 Disease caused by SARS-CoV-2 (disorder); SCTID: 840539006		Gabrielian et al [26] (tuberculosis) Mons [35] (SARS-CoV-2) Blomberg and Lauer [47] (SARS-CoV-2)	
	Clinical pharmacology (qualifier value); SCTID: 394600006	N/A		Celebi et al [33]	
	Child and adolescent psychiatry (qualifier value); SCTID: 394588006	N/A		Kassam-Adams et al [21]	
**Disease (disorder); SCTID: 64572001**					
	Degenerative disorder (disorder); SCTID: 362975008	Multiple sclerosis (disorder); SCTID: 2470000		Peeters [43]	
	Neurocognitive disorder (disorder); SCTID: 70907300	N/A		Zondergeld et al [36]	
	Genetic disease (disorder); SCTID: 782964007	Facioscapulohumeral muscular dystrophy (disorder); SCTID: 399091004		Guien et al [40]	
**Information systems**					
	Information system software (physical object); SCTID: 70659400	Cardiology information system application software (physical object); SCTID: 467522006 Emergency care information system application software (physical object); SCTID: 468401001 Radiology information system application software (physical object); SCTID: 464066007 Hospital administration information system application software (physical object); SCTID: 462944003		Kass-Hout et al [19] (cardiology) Bhatia et al [27] (emergency care) Kalendralis et al [37] (radiology) Haux and Knaup [31] (hospital administration)	
Administrative procedure (procedure); SCTID: 14734007			N/A		Haux and Knaup [31] Looten and Simon [39]
Infection surveillance (regime or therapy); SCTID: 170502008			N/A		van Panhuis et al [15] Mons [35]

^a^SCTID: SNOMED-CT identifier.

^b^N/A: not applicable.

### Approaches Used or Piloted in the Implementation of the FAIR Data Principles in the Health Data Domain Since the Conception of These Principles in 2014

#### Overview

A total of 50% (17/34) of the publications contained details on the approaches in use or under development in the implementation of the FAIR data principles.

We examined the approaches used in FAIRification and how each approach helped achieve a particular FAIR data principle. We then grouped similar approaches to better understand how the different approaches build upon each other. The publications we examined for this purpose can be found in Multimedia Appendix 2 [6,16-19,21-25,27,29,31-35,42,44,45,47].

#### Data Harmonization and Standardization

Approaches to data harmonization and standardization are key to facilitating data discoverability and reuse. Kassam-Adams et al [21] asserted that it is useful to distinguish between “standardization” and “harmonization” of variables drawn from multiple studies to facilitate data reuse. “Standardizing” has been defined as *establishing common variable names and response values for essentially identical data points collected in different studies* (eg, child age in years and values assigned to item responses within an established measure), whereas “harmonizing” has been defined as the *process of deriving a new common variable from existing data that measured the same or similar constructs* (eg, educational level as defined in different countries or intrusive thoughts about a traumatic event as assessed by different posttraumatic stress disorder symptom measures in 30/34, 88% of the studies from 5 countries) [21].

The American Heart Association Precision Medicine Platform aims to facilitate data findability through a transparent cloud-based platform with explicit harmonization approaches: identifying common parameters across all data sets and allowing forum users to interactively find or merge data of interest [19].

#### Data Dictionaries

Both Lacey et al [24] and Kassam-Adams et al [21] recognized data dictionaries as a critical step toward achieving data FAIRness in trauma studies and the California Teachers Study. A data dictionary is a centralized repository of data. It describes the content, format, and structure of a data set and conveys meaning, relationships to other data, origin, and use [49].

A case study on an emergency department catalog was conducted to FAIRify the emergency department data sets to improve data searchability. Interestingly, most data sets did not meet the requirements of this case study as they were not accompanied by a publicly available data dictionary [27]. The standardization of metadata has also been discussed by Kugler and Fitch [25] and Caufield et al [16]. We observed that standards for data publication need to be upgraded to require that a data dictionary accompanies every published data set [27].

#### Unique Identifiers for Data Objects

Navale et al [17] have discussed the role of digital object identifiers and globally unique identifiers in data findability and in patient deidentification in research studies. The role of unique identifiers in facilitating record linkage in Integrated Public Use Microdata Series (IPUMS) population surveys has also been discussed in the study by Kugler and Fitch [25], in which the unique identifiers link the same individual as they appear across multiple demographic studies. This has enabled researchers to study life courses [25].

#### FAIR Frameworks

Some tools have been developed based on the requirement analysis by multi-stakeholder efforts, such as the SCALEUS FAIR Data (SCALEUS-FD) tool [45]. SCALEUS-FD produced a specification for the description of data sets that meets key functional requirements, uses existing vocabularies, and is expressed using the Resource Description Framework (RDF). Similar work has also been discussed by Bhatia et al [27] and Dumontier et al [18], who presented the design of an open technological solution built upon the FAIRification process proposed by GO FAIR. Closing the gaps in this process for health data sets provides the health research community with a common, standards-based, legally compliant FAIRification workflow for health data management [18]. The actual implementation of the proposed architecture was initiated as an open-source activity, developing a set of software tools addressing different steps of the FAIRification workflow. GO FAIR also used the results from focus group discussions to gather requirements and a literature review of the GDPR and national legislations to architecturally design an open technological solution built upon the FAIRification process for multinational health data sets [6]. Finally, Suhr et al [29] described functional and quality requirements based on many years of experience implementing web portal data management for biomedical collaborative research centers.

#### Data Linkage and Semantic Web

Data linkage allows for the identification of the same individuals as they appear across multiple study cohorts even after pseudonymization [25]. Linked data refer to an ecosystem of technologies, recommendations, and standards that aim at the interconnection of heterogeneous data in 1 unified processing realm [50]. Many linked data (semantic web) standards and recommendations are based on RDF, which uses Uniform Resource Identifiers (URIs) to unambiguously identify data items. URIs make data uniquely identifiable and, thus, findable and accessible through the internet [51].

Natsiavas et al [44] listed RDF, RDF Schema, and the Web Ontology Language as the main languages used to define knowledge in the semantic web paradigm in pharmacovigilance work on OpenPVSignal. This enables both syntactic and semantic interoperability (SI) by defining the rules for communicating data, the semantic structures to represent knowledge, and the interlinking of data with third-party data sets or ontologies [44]. Schaaf et al [32] highlighted that SI is not considered in FAIR and showed that integrating metadata repositories into clinical registries to define data elements in the system is an important step toward unified documentation across multiple registries and overall interoperability.

#### Semantic Enrichment

We identified various studies (6/34, 18%) that discussed semantics and ontologies in the context of FAIR [26,32,33,35,47]. Mons [35] described the need for rich FAIR metadata to enable controlled, computational access for analysis or visualization as well as expert data annotation in the wake of the global COVID-19 crisis. European life-science infrastructure for biological information (ELIXIR) aims to ensure that FAIRified COVID-19 data are well annotated and accessible for reuse by the scientific research community, as well as providing a registry to collect COVID-19–related workflows [47]. Semantic modeling in pharmacovigilance was also described by Celebi et al [33] as a necessary activity comprising semantic harmonization and integration, requiring the reuse or creation of models compliant with the FAIR principles and requirements gathered.

#### Data Pseudonymization and Anonymization

Data pseudonymization and anonymization are steps taken to FAIRify data that are unique to the health research domain because of the sensitive nature of the data. Tools have been developed for this purpose [21,30]. We also found studies that highlighted the need for data owners to provide a description of the data pseudonymization method, especially where different methods are used to pseudonymize data [31]. The method of pseudonymization and anonymization is dependent on the purposes for which the collected data are intended. One-way encryption of identifiers, fuzzing, generalization, and longitudinal consistency are among the pseudonymization techniques that may be used. The Health Insurance Portability and Accountability Act deidentification standard of protected health information provides for the data types that should be erased from a health data set to minimize the risk of reidentification of data subjects [6]. None of these measures can ensure that the risk of reidentification is zero, and as pseudonymization and anonymization tools continue to develop, so do new technologies that facilitate brute-force attacks [6,52].

#### Use Case–Based Approach

It is also interesting to see the development of a template that includes instructions for writing FAIR-compliant systematic reviews of rare disease treatments. Doing so enables the assembly of a Treatabolome database that complements existing diagnostic and management support tools with treatment awareness data [38]. We found studies that discussed various aspects of FAIR repository design [22-25,36,42].

Efforts have been made to FAIRify a wide range of data that inform the management and prognosis of various diseases such as Huntington disease, cancer, posttraumatic stress disorder, and cardiovascular diseases. Although efforts to FAIRify data on a wider variety of diseases are ongoing, researchers have highlighted the need to FAIRify the scarce data on rare diseases and find and reuse the already existing data to benefit researchers, pharmaceutical companies, health care practitioners, and patients [32].

### IT Infrastructure, Workflows, and Tools for FAIRification

#### Overview

We identified 59% (20/34) of publications that described IT infrastructure, workflows, and tools for FAIRification.

Various workflows, tools, and infrastructures have been designed by groups worldwide to facilitate the FAIRification of health research data, as shown in Multimedia Appendix 3 [6,15-17,19,21,24,25,28,29,32,33,36,37,40,43-47]. However, the steps involved in the workflows, tools, and infrastructures vary. We also uncovered a wide range of problems and questions that researchers are trying to address by FAIRifying their data. We examined 4 workflow design purposes:

- To provide the health research community with a common, standards-based, legally compliant FAIRification workflow for health data management. The actual 10-step implementation of the proposed architecture has been initiated as an open-source activity for developing a set of software tools addressing different steps of the FAIRification workflow [6].
- To describe the 4-step FAIRification of a highly cited drug-repurposing workflow (OpenPREDICT) by FAIRiying data sets as well as applying semantic technologies to represent and store data on the versions of the general protocol, the concrete workflow instructions, and their execution traces [33].
- To describe a 4-step method to revolutionize the management of multiple sclerosis to a personalized, individualized, and precise level using FAIR data [43].
- To provide a 5-step guidance and give detailed instructions on how to write FAIR-compliant, homogeneous systematic reviews for rare disease treatments to facilitate the extraction of data sets that are easily transposable into machine-actionable information [38].

We found 11 tools designed for various purposes, as shown in Multimedia Appendix 3 [6,15-17,19,21,24,25,28,29,32,33,36, 37,40,43-47]. The Tycho 2.0 tool was instrumental in illustrating the value of investing in a domain-specific open-data resource for accelerating science and creating new global health knowledge through data FAIRification [15]. Another tool expands the value of clinical case reports as vital biomedical knowledge resources by structuring extensive metadata for clinical events and case descriptions. This standardization of metadata templates for clinical case reports aids clinicians and clinical researchers in gaining a better understanding of disease presentations, including their key symptomatology, diagnostic approaches, and treatment [16]. SCALEUS-FD is a semantic web tool that was built following the semantic web and linked data principles to support the difficulties of researchers in sharing their data by publishing FAIR-compliant data and metadata to facilitate interoperability and reuse [45]. Another tool was developed to present a novel ontology aiming to support the semantic enrichment and rigorous communication of pharmacovigilance signal information in a systematic way, focusing on the FAIR data principles and exploiting automatic reasoning capabilities on the interlinked pharmacovigilance signal report data [44]. This ontology uses RDF Schema and Web Ontology Language to define concepts as well as high-level semantic relations between them, as opposed to the free-text format based on which pharmacovigilance signal reports are made publicly available from organizations, which does not facilitate systematic search and automatic interlinking of information. Semantic web technologies and ontologies are key to the standardization and FAIRification of clinical data for training using machine learning algorithms that can be used to build prediction models for personalized therapy [34].

A FAIR data archive was built to better examine the nature and course of children’s responses to acute trauma exposure by combining data from multiple studies, describing key study- and participant-level variables, harmonizing key variables, and examining retention at follow-up across studies [21]. Another data archive in the form of a registry was developed to describe the steps toward the architectural extension and implementation of the FAIR data principles in the Open Source Registry for Rare Diseases via a web Federal Demonstration Partnership. The Federal Demonstration Partnership allows institutional data owners to give access to their data sets in a FAIR manner and can be integrated into a larger interoperable system. At the time of this authorship, the focus was on building a first prototype [32]. The second registry we found was the FAIR French National Registry for patients with facioscapulohumeral muscular dystrophy, whose original design allows for the strong involvement of both patients and physicians since its inception in 2013 [40].

Tools that facilitate collaborative efforts and data discoverability were also highlighted in our findings. Menoci is a modular web portal for data collection; experiment documentation; and data publication, sharing, and preservation in biomedical research projects [29]. This software focuses mainly on the collection and integration of data and the comprehensive documentation and workflow support of all steps, from planning to sharing and publishing. Another similar tool is the FAIR-based American Heart Association Precision Medicine Platform, whose goals are to democratize data, make it easy to search across orthogonal data sets, provide a secure workspace to leverage the power of cloud computing, and provide a forum for users to share insights. The tool thereby addresses the challenges researchers face when accessing large public data sets today in finding, accessing, downloading, and interpreting each poorly harmonized data set individually [19]. We also saw a portal that enables easy access to clinical, radiological, and genomic patient data and instantaneously executes multi-domain hypothesis creation and testing. This portal is applicable for medical training as well as clinical and research purposes [26].

It was interesting to learn that the infrastructures we found were designed for various purposes. In the clinical care area, we found a FAIR system designed to use clinical data elements for electronic data submission, processing, validation, and storage within designated repositories [17]. For research management purposes, we found a pipeline built for the creation of FAIR data integration infrastructure for data creation, storage, and processing [28]. We also found 3 infrastructures that were developed for epidemiology research. One is a FAIR cloud-based approach for storing, analyzing, and sharing cancer epidemiological cohort data in a common, secure, and shared environment adopted by the California Teachers Study in 2014 [24]. The other is a point-and-click website (ClinEpiDB) that supports third-party discovery and reuse of primary epidemiological research data by incorporating resources, tools, vocabularies, and infrastructure. It also facilitates access to and interrogation of high-quality, large-scale data sets, which enables collaboration and discovery that improves global health [46]. The third tool uses a model of data quality control and data stewardship that puts large and complex sensitive cohort data (YOUth) at the forefront of FAIR proper data infrastructure and management procedures [36].

We also found works conducted by the European life-science data infrastructure to facilitate collaborative research for improved access to research infrastructures and research data–sharing platforms in the EU in the wake of COVID-19 [47]. We noted that some investigators of the IPUMS data claim that their data collection approach was already consistent with the FAIR data principles before the FAIR concept was even conceived [25].

It is noteworthy that some of the reviewed FAIRification infrastructures, workflows, and tools have not been tested outside the pilot environment in which they were developed [6,45,47]. Thus, their applicability to real-world environments still needs to be demonstrated. Some of the built systems are also yet to be evaluated [34]. The development of some of the FAIRification tools and strategies has been based on the results of requirement-gathering exercises from the community [6,45].

#### Training

Our investigation revealed that comprehensive user training, including tutorials, conferences, or similar formats, is necessary to support the uptake of the FAIR principles as researchers continue to create or request FAIR data for reuse [25].

### Challenges and Mitigation Strategies in Approaches

#### Overview

A total of 38% (13/34) of the publications reported challenges faced or anticipated for the practical implementation of FAIR guiding principles to make their resources FAIR.

This section summarizes the reported challenges, risks, and mitigation strategies. It covers research questions 2 and 3 of our previously published protocol [13]. These are shown in Multimedia Appendix 4 [6,19,23,24,27,30,32,33,35,36,39,42, 47].

#### Findability

Löbe et al [30] pointed out several problems with the FAIRification of medical records. A challenge persists with determining a suitable granularity to which data should be assigned their own identifiers in cases in which external systems need to reference single data elements only. Löbe et al [30] also showed that frequent additions; updates to medical records; and subsequent changes, which include additions of data to the existing data, make iterative versioning a difficult task. Another reported challenge is the lack of “eternal persistence” of globally unique identifiers assigned by data repository software systems when updates or system changes are made. The authors also pointed out that there was no agreement on features such as descriptive human-readable identifiers as part of identifiers among digital identifier registry software, such as Health Level 7 Object Identifiers, digital object identifiers, and URIs.

Despite the high-quality metadata available for the YOUth study results, data are scattered in a Yoda file system confusion of folder titles, settings files, and headers in binary formats. To aggregate, validate, and store the metadata in an explicit metadata structure and facilitate mapping toward the Data Documentation Initiative standard, they developed a script in collaboration with the metadata specialist of the Utrecht University Library [36].

Findability challenges, such as the lack of tools to support content-based searching for data or catalogs without a data dictionary for a particular data set or a search function for a particular data element, were identified [27]. In these circumstances, users often have little insight into the content of the data unless they download the full data sets or follow links to the original data source, which can sometimes be broken. The inclusion of data dictionaries has been identified as a critical step toward improving data findability and accessibility, for example, the Emergency Department Catalog, a search tool designed to improve the “FAIRness” of electronic health databases [27].

#### Accessibility

Most of the challenges reported in the articles were accessibility-related. A long-standing, protective, and siloed research data management culture was reported as the main bottleneck for data sharing rather than the technical capabilities [23,24,30].

Cohort studies with stored data on local network drives, usually at the researchers’ institutions, create data silos that prevent real-time collaboration [24]. The manual work of merging, updating, and distributing individual and summary data sets for analysis and data sharing has been described as time-consuming and inefficient. Researchers have outlined that pivoting from every study investigator analyzing the copy of their data to all investigators using shared resources requires a conceptual shift in focus from the individual investigator to the broader user community [23,24]. To facilitate the cultural transformation of the workforce in biopharma, research and development envisioned a change from “it’s my lab and my data” to “it’s the company’s data” through incentives ranging from peer recognition to financial rewards [23]. A similar approach that entails engagement throughout the enterprise, from the departments to the executives, was also considered by Wise et al [23]. In this approach, a platform implemented through a combination of top-down commitment and investment from senior management and a bottom-up approach from scientists and managers has transformed their engagement and increased access to cohort data; removed many barriers to data reuse; and further accommodated data storage, cleaning, updating, analyzing, and sharing.

Cohort studies such as the YOUth study with a broad scope of data and potential interest to a broad range of researchers encounter frequent data requests [36]. The handling of a data request is a multistep procedure involving multiple actors. The authors highlighted that, by developing a system on the existing main data storage facility, they combined the request, staging, and transfer of data within a single system, which simplifies the process for all actors involved.

Regulatory burdens on data collection, such as data processing agreements, are also reported as challenges faced when handling data in diverse formats from different sources across different legal entities. A study on the impact analysis of the policy for access to administrative data in France reported that extrinsic factors influence the accessibility of claims data, such as human factors (eg, data scientists with experience in claims data) and economic factors (eg, data infrastructure that is Health Insurance Portability and Accountability Act– and GDPR-compliant) [39].

Data politics are shown to hinder access to “Real-World Observation” data. The Virus Outbreak Data Network Implementation Network (VODAN-IN) reported that the COVID-19 pandemic is highly politicized and that there is little chance that countries (or even institutions) will “share” their Real-World Observation data with even the World Health Organization [35]. The VODAN-IN sought collaborations with institutions that work on established knowledge bases and genuine partnerships worldwide, which has facilitated the enhancement of infrastructure and methods for “distributed deep learning” as a mitigation approach. Mons [35] also indicates that, based on the policy “as distributed as possible, as centralized as necessary,” the network strives to ensure that the algorithms and services can work effectively with both FAIR data and metadata.

The complexities of access to personal medical data lie in their sensitive nature for the individual patient [53]. The comprehensive GDPR sets several conditions and restrictions for data collection, including detailed consent research by stating the objective, the persons accessing the data, and the circumstances of data processing that prevent subsequent data sharing [30]. To fulfill GDPR requirements, developing and using further harmonized metadata vocabularies was suggested on topics such as the legal basis for data collection or the different variants of informed consent. Wise et al [23] also indicated that the GDPR compliance “right to be forgotten” should not be overlooked in the FAIR implementation plan. Wise et al [23] postulate that digital transformation will enable artificial intelligence analysis, machine learning, and data recognition.

Data owners’ privacy breaches during data sharing and safety concerns in cloud computing were reported as accessibility challenges [19,30]. Balancing the safekeeping of highly privacy-sensitive data and the protection of intellectual property and at the same time facilitating the scientific community’s access to these rich, unique data are among the reported challenges faced during data FAIRification by the American Heart Association Precision Medicine Platform and the YOUth longitudinal cohort study infrastructure in the Netherlands [19,36].

To mitigate community perceptions about the security of cloud computing environments, the American Heart Association platform has configured the cloud computing infrastructure, computation, and software platform with various security, confidentiality, and authentication settings to comply with widely adopted national and international standards and regulations. Another solution they have implemented is to have a third-party auditor assess the platform’s compatibility with national laws for analyzing biomedical data in a cloud-based environment, encrypt the data during transmission to or storage on the platform, and grant summary views of the results to the general community and detailed information to the other research group [19].

The fear of FAIR ecosystem monopolization is another concern that needs to be addressed. It is suggested that quality control and a minimal certification scheme for all components of the ecosystem must be in place as part of the effort to avoid monopolization of the FAIR ecosystem and its application by any particular party [35].

#### Interoperability

The interoperability challenge is reported in the scope of underuse of existing standards and lack of standard compliance in general. Löbe et al [30] pointed out that there are myriad standards, conventions, and best practices in biomedical research, but in many cases, researchers use the freedom of science to act on their own ideas rather than using existing standards. In contrast, data from health care systems are encoded with many different standards and governance models, and as a result, discovering, accessing, and linking such data is imperative in response to COVID-19 [47]. Moreover, the lack of domain-specific templates has been shown to force the use of a custom model, which limits interoperability [24].

The successful deployment of FAIR will require a standardized information architecture, and communities should reach a robust consensus on the ontologies they use to capture specific types of data. Löbe et al [30] argue that, although international medical terminologies such as the International Classification of Diseases, 10th Revision; Logical Observation Identifiers Names and Codes; or SNOMED-CT are very well suited to describe clinical concepts in detail, the vocabularies only partially fulfill the requirements of the FAIR data principles.

Furthermore, the terminologies overlap. Celebi et al [33] reported that creating a new ontology from scratch rather than creating a unified model based on the existing ontologies presents a challenge in the semantic modeling of unified workflow models. Regarding semantic modeling, Celebi et al [33] reported that the execution of the FAIRification process in the OpenPREDICT project was straightforward but the semantic modeling of the unified workflow model was challenging. Reusing existing semantic vocabularies to represent the unified model proved to be an extensive task. Moreover, some workflows have consistency issues such as missing joints and licensing elements, in addition to not conforming to the documentation intended for the specific project.

#### Reusability

Reusability is particularly challenging in the context of provenance; data quality; and enabling factors such as incentives, return on investment (ROI), and infrastructure. Provenance is a broad topic, and the demarcation of medical data acquisition is not sharp. Hence, data owners should detail the circumstances of data collection and processing (ie, data sources, data validation rules, format conversions, data cleaning, derived or aggregated data, measurement tools, scripts, software libraries, and observers). The provision of simple web-based visual analytics tools that give potential prospects an overview of the depth of available data can increase the reusability [30]. It is also recommended to develop robust distributed provenance information schemes that can balance full path reconstruction while keeping the process privacy compliant [42].

Data quality is an important concern when relying on external data. Very large quantities of data have been generated in relation to the global COVID-19 pandemic. Ensuring data quality and the risks of false and misleading information dissemination as “fact” was challenging [35]. Even though data donors are required to sign an agreement regarding the accuracy of the data they are sharing, the quality of the shared data is not always validated. Efforts made to ensure data quality through technical validations and manual maintenance should be explicitly mentioned [30,35]. The high quality of the metadata annotation was considered a key point for reuse. For example, data are published for broad access and reuse in many ELIXIR nodes, which aim to provide data management support to projects launched nationally and at the EU level [47].

Apart from the typical reusability challenges with regard to data quality and provenance, general financial and human factors (incentives) were reported as impeding factors for reusability. The uneven distribution of the effort and benefits of FAIRification at the expense of the data owners was also reported as a challenge. Data owners deserve an incentive for their efforts of sharing. Incentives may be required to support data sharing, enhance FAIR awareness through activities such as training, and promote methods to assess and support organizational and cultural shifts in management that lead to the achievement of positive perceptions of FAIR data sharing [30]. In the YOUth cohort success story, collaborative data management, by identifying executive and managerial key players in partner organizations; building good relationships; and adopting attitudes of patience, persistence, and forgiveness, helped overcome organizational differences and misunderstandings [36].

The costs of cultural and platform changes affect the key business categories of people, processes, technology, and data. More specifically, Lacey et al [24] described an up-front investment that was required to develop a data model, configure the user interface, and also convert decades’ worth of existing data sets into a single integrated data warehouse for data obtained from studies on large-scale cancer epidemiology cohorts. Although the initial costs of setting up FAIRification infrastructures and tools are quite significant, the costs of maintaining the infrastructure and tools once the initial setup is completed are much lower [24,54]. Executive management will need to be convinced that, apart from being a high-priority, urgent endeavor, FAIR implementation will generate a long-term ROI. Differing levels of uptake of FAIRification concepts among researchers may be observed during the transition [23,24].

**Figure 2:**
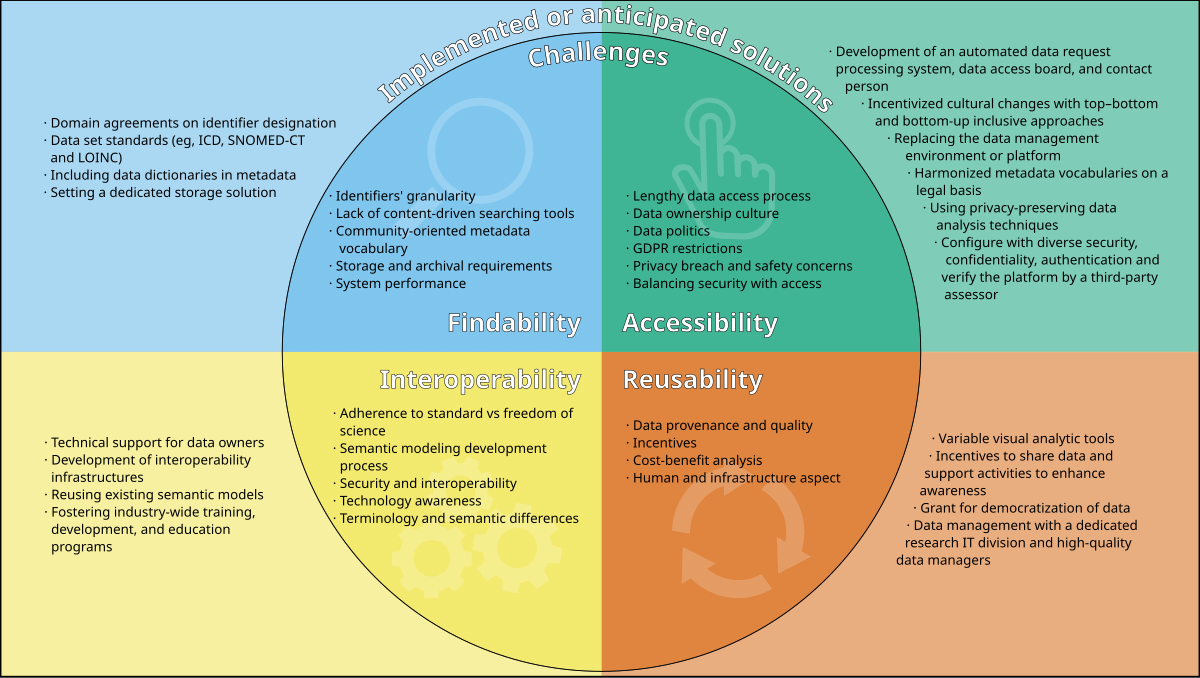
Summary of the challenges and mitigation strategies used in the implementation of the findable, accessible, interoperable, and reusable data principles in health data stewardship. GDPR: General Data Protection Regulation; ICD: International Classification of Diseases; IT: information technology; LOINC: Logical Observation Identifiers Names and Codes; SNOMED-CT: Systematized Nomenclature of Medicine–Clinical Terms.

Partner organizations with a clear ambition to support proper data management and accessibility and the means and institutional support to realize this ambition by providing a dedicated research IT division and high-quality data managers was a crucial prerequisite to the success claimed by the YOUth project [36]. Figure 2 presents a summary of the challenges and mitigation strategies used in the implementation of FAIR data principles in health data stewardship.

### Networks Involved in the Implementation of the FAIR Data Principles

#### Overview

A total of 35% (12/34) of the publications contained details on networks involved in the implementation of the FAIR data principles. The operational definition of “network” for this scoping review is “scientific communities, research institutions, repositories or data archives, consortia, funding agencies, and citizens who are actively engaged in advocating FAIR principle data stewardship in the health care domains.” Multimedia Appendix 5 [15,18-20,24,29,35,42,47] provides an overview of these networks.

Most continents have been reached by at least one FAIR network. All the networks we observed had different sources of funding. A common theme is the community approach through collaboration with parties sharing similar interests, such as the VODAN-IN, which was established to provide a platform for FAIR data exchange during the COVID-19 pandemic and a reference point for data stewardship in future pandemics [35]. The Research Data Alliance (RDA) is a community-driven initiative that was established in 2013 and consists of several work groups whose overall aim is to FAIRify health research data [55]. The RDA also established a working group to support the VODAN-IN [35].

Another network with a similar approach is ELIXIR, which has documented willingness to collaborate with appropriate stakeholders to facilitate discovery, access, and data linkage. ELIXIR has also expressed a willingness to further the development and application of normalization and interoperability of well-annotated medical and real-world data by seeking collaborations with researcher-driven initiatives by developing open reproducible tools and workflows for COVID-19 research [47]. Both networks are explicitly seeking collaborations with researchers who share similar interests.

A challenge facing COVID-19 research is that data from health care systems are encoded using many different standards and governance models. ELIXIR’s response aims to ensure that COVID-19 data are well annotated and accessible for reuse by the research community and society [47]. They have created databases and archives in which researchers are encouraged to deposit and share their raw sequence data and COVID-19 data in a manner compliant with the EU GDPR. Other networks that have highlighted the need for a communal approach in FAIRification endeavors are the World Wide Web Consortium Semantic Web for Health Care and Life Sciences Interest Group and the RDA [18,36].

The active networks have also identified challenges faced by data-driven research. The American Heart Association established the Precision Medicine Platform to address the current challenges in accessing large public data sets—lack of harmonization across multiple data creates difficulties for researchers to combine data sources and evaluate results. The aim is to provide a transparent and explicit harmonization to access both harmonized and raw data [19]. Although not yet completed, this network plans to involve the community for better data diversification. Similarly, the Biobanking and BioMolecular resources Research Infrastructure–European Research Infrastructure Consortium extended the FAIR principles to FAIR health principles in biological material management by providing comprehensive provenance information for the complete chain, from donor to biological material to data, as well as incentives for enriching existing resources and reusing them [42].

The Dutch Research Council, Data Archiving and Network Services organization, and UK Data Service have come together to develop a high-quality research data infrastructure for sensitive cohort data [36].

The VODAN-IN welcomed organizations that work on established knowledge bases and biomedical research results for collaboration in FAIRification efforts [35]. The VODAN-IN has a long-term goal of reusing the resulting data and service infrastructure for future outbreaks. Similarly, some of the active networks such as the Committee on Data for Science and Technology have indicated that their areas of future research will apply data to real-world issues and promote the application of principles, policies, and practices that enable open data and advanced data skills for national science systems. Major discussion points in these data-sharing networks are data discoverability, infrastructure development, infrastructure deployment, and collaborative efforts in the FAIRification journey [15,18,20,24,29,33,36,47].

With respect to future work, the World Wide Web Consortium Semantic Web for Health Care and Life Sciences Interest Group has indicated interest in adding new use cases and documenting improvements made to the existing community profile [18]. Similarly, the American Heart Association has identified the need for better data diversification and has plans to involve the community in this venture [19].

In the same community spirit, FAIRsharing intends to grow the number of users, adopters, collaborators, and activities, all working in their community-driven resources to enable the FAIRification of standards, knowledge bases, repositories, and data policies [29].

The VODAN-IN has identified the need to provide a platform for collaborative FAIR data exchange during the COVID-19 pandemic, becoming a reference point for data stewardship in future pandemics [35]. DataMed’s goal is to be for data what PubMed has been for scientific literature by enabling the discovery of biomedical data sets that are spread across different databases and on the cloud [20]. The Sherlock Division at the San Diego Supercomputer Center has identified the need to expand the geospatial, comorbidity, and biospecimen tools for query and analysis; automate certain processes; and develop an application programming interface enabling data collection and sharing [24].

### Outcomes of FAIRification and Expected Future Work

#### Overview

A total of 68% (23/34) of the publications contained details on the outcomes of FAIRification endeavors and the expected future work.

Several outcomes were reported or expected by researchers as a result of attempting to incorporate the FAIR guiding principles into their specific use cases. We categorized the outcomes into new findings or treatments, improved data sharing and publications, and ROI, as shown in Multimedia Appendix 6 [15,16,21-27,29-34,36,40,41,43-47]. On the basis of the research findings, we identified the principles needed to achieve the outcomes and the relevant future work that the authors indicated.

#### New Findings or Treatments

In frontline clinical care, rich metadata acquired from clinical case reports serves as a valuable tool empowering researchers to explore disease progression and therapies to improve health outcomes [16]. The effort to enrich data with explicit metadata using standardized templates has also facilitated wider data provision for educational documentation. The data can be easily identified by metadata, reducing the time spent collecting and searching for already available data. The standardization of metadata templates reduces the time and costs of data discovery. These savings lead to improved productivity in health research and development [23]. Tools such as the Tuberculosis Data Exploration Portal allow for collaborative research that uses the wealth of metadata contained within the database to improve patient care, especially in the case of drug-resistant tuberculosis [26].

#### Data Sharing and Publications

At the corporate level, implementing the FAIR principles improves robotics and process automation through machine readability, which further enables reuse and scalability [23]. The difficulties experienced by researchers when sharing their data have led to the creation of tools that lighten the burden of publishing interoperable and reusable data and metadata [45]. Successful standardization and harmonization of data and metadata in clinical tools such as registries have enhanced data-sharing capabilities. Time will show if disease registries can simultaneously be FAIR and data privacy compliant in the wake of the GDPR implementation [32,45]. The combination of ontologies and semantic web has also allowed for FAIR clinical data sharing for the secondary use of administrative claims data [31]. In the field of radiation oncology, the radiation oncology ontology and semantic web have proven usable for integrating and querying data from different relational databases for data analysis [34].

FAIRification can also provide insights into understudied but clinically relevant matters such as pediatric traumatic stress. A sustainable framework for standard variable names, metadata, and harmonization algorithms has been developed to support data reuse [21]. The findability and accessibility of this archive have allowed investigators worldwide to reuse the published data [21]. More analyses are expected to be built on this accessible, reusable, harmonized child trauma data set. The research produced by IPUMS users, published as numerous articles, books, and papers, is abundant on Google Scholar. The pace of IPUMS-based publications continues to accelerate [25]. Publications that expand the notions of FAIR and FAIRification from the relatively static artifacts of data sets to publications on the dynamic processes of workflows have resulted from the FAIRification process [33].

A database in clinical epidemiology has been developed as an open-access web-based tool that maps data to common ontologies and further creates a unified semantic framework. The terms are reused or requested from existing ontologies when possible. The context of these terms is provided to facilitate reusability [46]. Once information related to pharmacovigilance safety signals is identified, it is publicly communicated in free-text form by organizations charged with the responsibility of identifying the safety signals for further investigation [56]. The OpenPVSignal ontology was developed to support the semantic enrichment and rigorous communication of pharmacovigilance signal information in a FAIR manner by use of existing semantic-rich metadata. It also interlinks the respective information with other data sources by applying semantic reasoning, which further enables data reuse [44].

The improvements made in Project Tycho, version 1, as a result of FAIRification gave rise to Project Tycho, version 2, demonstrating the value of sharing historical epidemiological data for creating new knowledge and technology. This has also facilitated data reuse [15]. These improvements resulted in 150 published works that cited the Project Tycho release paper, 47 of which were published by authors from 1 of the 100 institutions most commonly affiliated as registered Project Tycho users [15]. A tool has been developed to facilitate the collection of harmonized, rich data and metadata as well as the standardization and documentation of experimental data along the scientific process. This tool also allows for data sharing with public repositories. Data access is regulated by the Drupal framework [25].

The American National Cancer Institute established a framework to protect the security of cancer data warehouses under its jurisdiction [57]. We noted that, although the infrastructure of the data warehouse of the California Teachers Study has unique features in line with this framework, it still shares enough common characteristics to facilitate widespread data harmonization, pooling, and sharing. The California Teachers Study now offers all users a shared and secure workspace with common data, documentation, software, and analytic tools to facilitate use, reuse, and data sharing [24]. The combination of ontologies and semantic web technologies has also been shown to enable FAIRification of clinical data, which further facilitates data sharing [31,34]. However, our review led us to resonate with previous studies that have highlighted the need to evaluate data access policies to understand the potential leverages of data reuse [39].

#### ROI Outcomes

ROI remains an open discussion [22-24,36]. Digitizing and standardizing all data is more expensive than digitizing a specific section of interest. In contrast, simultaneously digitizing and standardizing data is more efficient and budget-friendly than iteratively doing the same work for small parts of the data at a time [15]. Studies also show that poor adherence to the FAIR data principles hampers data use and reuse, but this is correctable at a reasonable cost [41]. Additional investment in human resources may be required for the purposes of preparing the data for FAIRification, actual FAIRification, and related activities. Once complete, FAIRification will prevent duplication and accelerate new science and discovery in global health [15]. The short-term impact of FAIRification includes improved data findability; faster data access; and the selection of standardized, machine-readable data for analytics [23]. The reusability of the data increases their value as different researchers worldwide continue to request these data and may eliminate the need for a new process of data collection [21,25].

Some of the FAIRification efforts have delivered with regard to ROI. Retrospective FAIRification of data and infrastructure may require a significant up-front investment to develop models and prepare data sets that may have been collected and maintained over the years for FAIRification. However, as increasingly more options and examples for FAIRification become available, the required up-front investment may decrease. As FAIR health data are still a relatively new concept in the field of health, it is critical that community members share their experiences, perspectives, and lessons learned in their FAIRification endeavors. There is also need for further research that will provide insights into the organizational, behavioral, and technical shifts that occur as a result of the transitions to FAIR [24].

#### Future Work

Collaboration with appropriate stakeholders has been described as a key enabler for successful FAIRification efforts [47]. Active networks repeatedly mention plans to collaborate with appropriate stakeholders to interconnect the different researcher-driven initiatives, include FAIRified data sets from a wider variety of sources, and develop open reproducible tools and workflows for research [27,47]. Future work will also involve expanding tools to allow for automatic semantic data retrieval and integration with third-party tools and systems [29]. The performance of these systems may also need to be evaluated [41]. A notorious challenge facing FAIRification is the high costs and effort required for the successful conduction of data collection [30]. In the future, it may be astute to use incentives to motivate stakeholders to take on the FAIRification journey. We may also see a rise in the standardization of processes for data access and extraction in the future [30]. There are plans to further develop the repositories in use so that they can accommodate data sets from a wider variety of sources [27]. There are also efforts to develop public catalogs that promote external metadata discoverability through which researchers worldwide can access detailed metadata [36]. There may be a need to research potential conflicts of interest that may arise among the various stakeholders in the wake of FAIR health research data sharing, as well as the measures to mitigate unethical use [41].

## Discussion

### Principal Findings

#### Overview

We appreciate the efforts that the various reviewed authors have taken to implement the FAIR data principles in health research data. The overall concept of data management, of course, is not new to the research ecosystem. Unlike other initiatives that focus on human scientists, the FAIR principles emphasize improving the ability of machines to find and use data automatically and supporting its reuse by individuals [35].

Critical steps toward data FAIRification in the biomedical domain include interpolation, inclusion of comprehensive data dictionaries, repository design, SI, ontologies, data quality, linked data, and record linkage, as well as requirement gathering for FAIRification tools. Other concerns such as pseudonymization, the need for collaboration in matters of FAIRification, and data FAIRification in the wake of COVID-19 were discussed. In conducting this study, we also noted the varying levels of understanding and misunderstanding of the FAIR concept. In this section, we discuss 7 recurring themes in our findings.

#### Requirement Gathering

Involving the intended users in requirement engineering during the development of software tools is a vital first step in ensuring both tool acceptability and applicability [58]. Requirement gathering for FAIRification tools and strategies helps deal with the sense of mistrust within the community when it comes to matters of data safety and privacy. It may be necessary to encourage community participation by first making the community aware of the potential benefits of FAIRification as well as the steps taken to ensure the safety and privacy of their data with regard to legal regulations.

#### Data Dictionaries

The importance of a comprehensive data dictionary for data sharing cannot be overstated [59]. Data dictionaries provide context for data collection, documentation, conversion processes, generation, validation, storage, and use [49]. Standardized data dictionaries lead to more efficient data handling and analysis, which further improves data interoperability [60]. Data dictionaries also enhance a user’s understanding of the data and, therefore, help improve data reusability [49]. The inclusion of data dictionaries is a critical step toward improving data accessibility and overall FAIRness [21,24,61].

#### Legislation

There is a need for legislation in matters of FAIRification of sensitive health data [62]. According to the GDPR, patients own their data. This is currently factored in during FAIRification processes in the EU and higher-income countries. However, there are countries where legal frameworks regarding data governance have not yet been established [63]. Rapid advances in technology allow huge amounts of health data to be collected and manipulated within a short time. Consequently, more comprehensive data protection strategies are needed. However, what is the ethical route to take in the event that the law regarding data sharing is neither permissive nor explicit? What will govern FAIRification or FAIR data sharing in these instances? Is now the time to have a conversation on law amendments that may allow for more FAIRification in a protected environment? What are the restraints if granting agencies and funders of research require open data sharing? The answers to these questions may facilitate voluntary FAIR sharing of health data for purposes of coordinating responses to health threats, health research for better prognosis, surveillance, policy making, and decision-making.

#### Clinical Registries

The data maintained in clinical registries play a vital role in patient management, research, policy, and decision-making [64]. This is especially critical in the domain of rare diseases, where resources are scarce [38]. It is equally important that the data be linked using similar terminologies for the data values and data types to enable data compatibility [65]. This eliminates the need to recollect data and reduces the clerical burden that researchers have of resolving incompatibilities and correcting errors. It also allows machines to aid in data analysis across resources as they depend on explicit, unambiguous definitions of data [40].

#### Required Organizational Cultural Shift

In the course of conducting this study, we observed the immense effort and time required to FAIRify data and metadata retrospectively [32,45]. It is for these reasons that we reiterate that the concept of FAIR data stewardship should be considered in the early stages of a project. However, this will require significant cultural and organizational changes that may be met with resistance from the various stakeholders involved. For example, semantic enrichment of health data at the source is a critical part of data FAIRification, but studies show that frontline health professionals are still not motivated to add the process of semantic enrichment to their workflows [21]. Therefore, it is necessary to find innovative ways to add this step and the related technology to the workflows that already exist while incentivizing health care professionals to do the same.

#### Semantics

SI allows data generated in different systems to be interchangeable with consistent meaning [66]. This is a foundational aspect that determines the ability of different systems to effectively work together and share data and domain concepts, context knowledge, and formal data representation [67]. However, application of the FAIR principles does not guarantee SI, and it is in this vein that Natsiavas et al [44] suggested that it would be astute for the global health community to establish a collection of preferred standards and ontologies. In the course of this review, we came across studies that attempted to include SI in a registry designed for rare diseases. A metadata repository offered possible uniform descriptions and defined data elements, creating SI in a FAIR infrastructure, which further facilitates data findability, sharing, and reuse [32].

#### Training on FAIR

FAIRified clinical case reports have also been shown to be an attractive educational resource to a larger diverse audience if enriched with standardized metadata [16].

Several training platforms target FAIR education. The Precision Medicine Platform, for example, provides tutorials that guide researchers through data analyses such as genome-wide association, population demographics, descriptive statistics, and deep learning [19]. IPUMS supports FAIR through an extensive program of user training and support in the form of brief video tutorials on the use of the data extraction systems. In-person training and workshops at conferences, as well as active user forums, complement the portfolio [25]. Are these training workshops effective?

### Authors’ Reactions

We recognize that it is probably not entirely practical to have a single template or workflow that guarantees an outcome of FAIRified data. Many technical and administrative questions must be asked about influences on the FAIRification process.

Is the *required expertise* for this process available? Are there enough time and finances to train a select few to become FAIR data stewards within the organization with the intent of nurturing skills and expertise within the organization, for example, by implementing a *train the trainer* concept [68]? Do time and financial constraints necessitate that it is better to outsource FAIR expertise? What is the *budget available* for this process? As FAIRification is an iterative continuous process, what is the long-term plan to *iteratively improve the FAIRness* of the data? As there is more than one way to evaluate FAIR, what method of evaluation should be chosen in a particular context or data domain and why? What if the evaluation method is not applicable to the analysis of some of the software tools used, as is the case with the FAIR metrics [28]? Is the *data management plan* FAIR-inclusive? What is the purpose of FAIRification for this specific context? For the purposes of meeting the specific objectives of FAIRification, which of the 15 FAIR subprinciples must be adhered to in said context, and which ones can be left out [69]?

Although many benefits have been demonstrated in the FAIR work that we have reviewed, the related efforts, time required, and costs may be above reach for many researchers. Incentives may be needed to motivate stakeholders to embrace the FAIRification journey [30]. Subsidizing the costs involved in data FAIRification may also prove to be a worthy investment [36]. Still, questions may arise regarding the expected ROI for all the participants involved in this process and the legal constraints, implications, or risks that need to be navigated.

The development of the FAIR principles in clinical research may serve as an important step toward the standardization of data elements, but it still does not guarantee data users a concrete minimum viable product. However, at the same time, FAIRification efforts are expected to be iterative, but to what point? There may be a need for the various stakeholders involved to define the minimum viable product.

There may also be a need to discuss the sustainability of the developed tools and infrastructures for FAIRification as well as the FAIRification outcomes. Further discussion is needed on the expertise required to operate software to realistically prepare the users for these tools and infrastructures. For example, our further study of the work done by Caufield et al [16] on using FAIRshake (to evaluate the FAIRness of the metadata acquired from clinical case reports) led us to discover that FAIRshake users need to be efficient Python programmers. It is also worth discussing how the required funding and human resources are maintained over time.

### What FAIR Is Not

Our work led us to realize the need to clarify the things that FAIR is not. These include those outlined in the following sections.

#### Open

FAIR and open data are not the same; they are 2 distinct concepts. However, they are becoming increasingly close. “FAIR” and “open” represent 2 different concepts, and the 2 words cannot be used interchangeably to mean the same thing. We found studies in which these 2 distinct concepts seemed to mean the same thing [19]. FAIR data management does enable more shared research results from open publications.

#### The Pathway to Better Data Quality

We observed that some authors claimed that the FAIRification of their data led to an increased quality in the data available for analytics, such as machine learning [23]. Previously conducted studies have shown that, conceptually, this is not the case [70]. However, a study in our review claimed that the data quality and consistency of submissions were enhanced through validation with domain-specific data dictionaries. This claim may still require further investigation.

#### Advertising

We also observed that authors claimed that they achieved findability as their data resources were actively promoted by the staff of the organization as well as by happy users in various forums, such as workshops and conference presentations, publications, classrooms, blogs, and social media [25]. Further evaluation of these claims is required to determine how the findability principle in FAIR is met.

#### Free

In our review, we identified studies that stated that the data were accessible as they were provided free of charge and with as few restrictions as possible [25]. However, Mons [35] indicated that free does not mean FAIR. Further investigations are needed to investigate these claims [35].

#### User-Friendly

We observed that some authors claimed that they improved the findability of their data by carefully designing the user interface to ensure that users could navigate large data collections to locate the specific data they needed for their research [25]. The same authors claimed that the data were accessible as the tool’s interface was easy to use, which allowed users to navigate their large data collections to access free data with as few restrictions as possible. There remain more criteria to be fulfilled before claiming data findability or accessibility.

### Strengths and Limitations of This Study

We used independent reviews of 3 databases throughout the extraction phase. However, we were not able to critically examine the gray literature as it was focused on specific health research domains; therefore, it did not meet our inclusion criteria. Our strict focus on the health domain may have led us to miss out on other important developments, collaborative data approaches, and data-sharing initiatives in data FAIRification for intersecting domains. Many of the reviewed FAIRification efforts did not provide an objective FAIR evaluation. Therefore, it is not entirely possible for us to have an actual score that depicts the extent to which each of the 15 FAIR subprinciples was fulfilled [69].

This review covers work until the end of 2020. We expect a sharp rise in publications owing to the relevance of the topic in the wake of the COVID-19 pandemic. Therefore, it may be valuable to further extend this work to a systematic review covering the time range until the present. An evaluation of the quality of the publications remains a potential factor in judging the statements extracted from the included publications. Further research is needed to evaluate the quality of the included papers as well as the quality of the application of the FAIR principles in the included publications.

### Conclusions

This work brings together a series of initiatives, concepts, and implementation practices of the FAIR data principles in health data stewardship practices. The results of this review are useful in identifying gaps and further areas of research. We identified aspects of FAIR that seem to be misunderstood, and we recommend further training on them. We hope that this work will serve to inform decisions on the FAIRification journey and provide a comprehensive introduction to the various stakeholders who may want to know what to anticipate before embarking on the FAIRification journey. We also anticipate that this work will be of value in informing decisions on the readiness of research institutions to embark on the FAIRification journey. This work may also serve as a valuable tool for the developers of FAIRification tools and infrastructures as they strive to meet the needs of the stakeholders involved. It would be interesting to conduct further studies on the reproducibility of the FAIRification assessment results. We recommend that solutions to the challenges and risks encountered be further investigated.

The implementation of the FAIR principles carries the promise of improved data management and governance through improved data sharing, standardization, harmonization, and deduplication of work [24]. The addition of rich metadata in the process of FAIRifying data has facilitated the discovery of said data, thereby increasing the audience. FAIR and open databases allow continuously updated data to serve as a valuable educational resource for clinical investigators, facilitating the development of novel advances in medical science and improved patient care [16]. All these examples contribute to a worthy ROI. In 2019, the cost of not having FAIR research data in the EU research economy as a whole was estimated to be €10.2 billion (a currency exchange rate of €1=US $1.07 is applicable) per annum, with more specific estimation expected to vary from domain to domain [71]. The interesting question is what are the costs of not having FAIR health research data now, and how much can be saved from the implementation of the FAIR data principles in this domain?

## Appendix

Multimedia Appendix 1 PubMed search strategy.

Multimedia Appendix 2 Approaches being used and piloted in the implementation of the findable, accessible, interoperable, and reusable data principles in the health data domain since 2014.

Multimedia Appendix 3 FAIRification IT infrastructure, workflows, and tools in use or under development.

Multimedia Appendix 4 Challenges and mitigation strategies with regard to the approaches used in the practical implementation of the findable, accessible, interoperable, and reusable data principles in health data.

Multimedia Appendix 5 Active networks involved in findable, accessible, interoperable, and reusable data principle implementation in the health data domain.

Multimedia Appendix 6 Outcomes and future work.

Multimedia Appendix 7 PRISMA-ScR (Preferred Reporting Items for Systematic reviews and Meta-Analyses extension for Scoping Reviews) checklist.

## Abbreviations

ELIXIR: European life-science infrastructure for biological information
EU: European Union
FAIR: findable, accessible, interoperable, reusable
GDPR: General Data Protection Regulation
IPUMS: Integrated Public Use Microdata Series
MeSH: Medical Subject Headings
PRISMA: Preferred Reporting Items for Systematic Reviews and Meta-Analyses
PRISMA-ScR: Preferred Reporting Items for Systematic Reviews and Meta-Analyses extension for Scoping Reviews
RDA: Research Data Alliance
RDF: Resource Description Framework
ROI: return on investment
SCALEUS-FD: SCALEUS findable, accessible, interoperable, and reusable data
SI: semantic interoperability
SNOMED-CT: Systematized Nomenclature of Medicine–Clinical Terms
URI: Uniform Resource Identifier
VODAN-IN: Virus Outbreak Data Network Implementation Network
