# Supplementary material for: Initiatives, Concepts, and Implementation Practices of the Findable, Accessible, Interoperable, and Reusable Data Principles in Health Data Stewardship: Scoping Review
Source: J Med Internet Res. 2023 Aug 28;25:e45013. doi: 10.2196/45013 (PMC10495848; doi:10.2196/45013)
Supplement: Multimedia Appendix 1 [file jmir_v25i1e45013_app1.docx]

((((("biomedical research") OR ("biomedical research"[MeSH]) OR ("clinical trial") OR ("clinical study"[Publication type]) OR ("Meta-Analysis as Topic"[MeSH]) OR ("Clinical research") OR ("Clinical records") OR ("Epidemiologic Methods"[MeSH]) OR ("Disease surveillance") OR ("Electronic Health Records") OR ("Electronic Health Records"[MeSH]) OR ("Medical research") OR ("Medicine") OR ("Nursing") OR ("Nursing"[Mesh]) OR ("pharmaceutical") OR (((("Pharmacy"[MeSH Terms] OR "databases, pharmaceutical"[MeSH Terms]) OR "Pharmaceutical Services"[MeSH Terms]) OR (("Pharmaceutical Research"[MeSH Terms] OR "evidence-based pharmacy practice"[MeSH Terms]) OR "pharmacy service, hospital"[MeSH Terms])) OR "Pharmacoepidemiology"[MeSH Terms]) OR ("public health"[All Fields]) OR ((((("Public Health"[Mesh]) OR "Nurses, Public Health"[Mesh]) OR "Public Health Surveillance"[Mesh]) OR "Public Health Informatics"[Mesh]) OR ( "Public Health Practice"[Mesh] OR "Public Health Nursing"[Mesh] OR "Public Health Systems Research"[Mesh] ))) AND ((Health) OR ("Healthcare") OR ("Health services research") OR ("Health services research"[MeSH]) OR ("delivery of health care") OR ("delivery of health care"[MeSH]) OR ("health research") OR ("outcome assessment, health care"[MeSH]) OR ("outcome assess*"))) AND (("Research") OR ("Data curation"[MeSH]) OR ("OPEN data") OR ("open repositor*") OR ("data curation") OR ("Data curation"[MeSH]) OR ("Data sharing") OR ("Information dissemination") OR ("Open publishing") OR ("Open access publishing"))) AND (("FAIR data Principles"[tiab]) OR ("FAIR metrics") OR ("FAIR principles") OR ("FAIR guiding principles") OR ("Data stewardship") OR ("Data management systems") OR ("Data management"[MeSH])))
