# Supplementary material for: Initiatives, Concepts, and Implementation Practices of the Findable, Accessible, Interoperable, and Reusable Data Principles in Health Data Stewardship: Scoping Review
Source: J Med Internet Res. 2023 Aug 28;25:e45013. doi: 10.2196/45013 (PMC10495848; doi:10.2196/45013)
Supplement: Multimedia Appendix 2 [file jmir_v25i1e45013_app2.docx]

| **Concept / approach** | **Contributing to FAIR principle** | **Approaches** |
| --- | --- | --- |
| Data harmonisation | R | Naming conventions for key variables [21]. |
| Data dictionary | F, A, I, R | Using a data dictionary to improve data interoperability, enhancing user understanding of data and thus improve data reusability, which is an issue that still plagues the research community [21,24]. |
|  | F, A, I, R | The EDCat system improved the overall FAIRness of HCUP-SEDD by adding standardized data dictionaries which serve to improve the findability of individual datasets and increasing the efficacy of searches for specific data elements and data types. A search tool was developed to find these datasets [27]. |
| Data standardisation vs data harmonisation | I, R | Standardizing is establishing common variable names and response values for essentially identical data points collected in different studies. Harmonizing is process of deriving a new common variable from existing data that measured the same or similar constructs [21]. |
| Standardization | F, R | Established variable naming conventions for key demographic, event, and symptom measure variables; these standard variable names are applied to each dataset upon submission. Future re-use of these datasets will require flexibility in how the timing of each assessment is considered. Therefore a common information across datasets about assessment timing at both the study and the participant level is provided as well as harmonized demographic data and symptoms of post-traumatic stress disorder [21]. |
| Data Harmonization and Searching | F | Helps data harmonization and searching challenges by identifying common parameters across all data sets and thus allowing users to interactively find or merge data of interest. Data harmonization on the platform is transparent, providing insight into how each data variable is defined. Filters and multiple graphics display the distributions of important clinical outcomes and risk factors and provide summaries of available data sets, allowing users to quickly identify a sub-cohort that meets their research needs. Users have access to both harmonized and raw data [19]. |
| Requirements gathering | F, A, I, R | Requirements suggested for the system to meet: should be a standalone application with simple configuration setting for typical non-IT users; must be self-describing where the solution must be by itself a FAIR object in the FAIR ecosystem; must be possible to store and describe multiple datasets; should make the data FAIRer; must expose its services over the web; for the authorization, all users can access the metadata; should have a user-friendly interface besides being a piece of machine-actionable software; must allow data queries compliance with a widely used standard query language [45]. |
|  | F, A, I, R | This work describes the results of a multi-stakeholder effort to produce a specification for the description of datasets that meets key functional requirements, uses existing vocabularies and is expressed using RDF. This includes provenance and versioning, retrieval and reuse [18]. |
|  | F, A, I, R | This work describes the methodology to publish scientific workflows as FAIR data. It includes structured interviews with data scientists and a gap analysis of the literature, to formulate user requirements for the reproducibility of workflows [33]. |
|  | F, A, I, R | This work presents the architecture design of an open technological solution built upon the FAIRification process proposed by GO FAIR closing the gaps of this process for health datasets, thus providing the health research community with a common, standards-based, legally-compliant FAIRification workflow for health data management. The actual implementation of the proposed architecture has been initiated as an open-source activity (<https://github.com/fair4health>) [6]. |
|  |  | This work describes the functional and quality requirements based on many years of experience implementing data management  including: PIDs and descriptive metadata should be integrated into routine workflows and information systems in biomedical research should be a central goal for infrastructural software development; upload of annotated research data to public repositories as required by some journals; Utilization of Handle system PIDs for assets and datasets, display and download of machine-readable metadata, and use of the open HTTP(S) protocol are important measures to achieve findability and accessibility; Data access control is consistently enforced through the Drupal framework. Some criteria focus on documentation and implementation on a per-instance basis rather than on functionality of the software; Interoperability and reuse of data are partly supported where semantic JSON-LD mark-up of collected information is accessible or data is replicated into public data repositories [29]. |
| Semantic Interoperability | I | Semantic interoperability is not considered in FAIR. This tool offers a possibility to uniformly describe and define data elements with a metadata repository that is used as an additional tool for creating semantic interoperability in a FAIR infrastructure [32]. |
| Linked Data | F, I | Linked data is an ecosystem of technologies, recommendations and standards which interconnect heterogeneous data. Linked Data standards and recommendations are based on RDF which uses URIs to unambiguously identify resources [44]. |
| Enabling syntactic and semantic interoperability | I | RDF, RDF Schema, and the Web Ontology Language (OWL)—the main “languages” used to define knowledge in the Semantic Web paradigm—enable both syntactic and semantic interoperability by defining the rules for communicating data, the semantic structures to represent knowledge, and the interlinking of data with third-party datasets or ontologies [44]. |
|  | F, A, I, R | This work demonstrates how a novel ontology supports the semantic enrichment and rigorous communication of pharmacovigilance signals by publishing FAIR signal information and exploiting automatic reasoning capabilities upon the interlinked pharmacovigilance signal report data. This ontology is built upon a reusable, extendable and machine-understandable model based on the Semantic Web standards/recommendations. can therefore facilitate heterogeneous data interlinking, semantic and syntactic interoperability, provenance tracking and knowledge expressiveness [44]. |
|  | F, A, I, R | This work shows the development of an ontology to be used in the field of radiation oncology to map clinical data from relational databases. The ontology was combined with semantic Web techniques to publish mapped data and easily query them using SPARQL. The combination of these clinical FAIR data sources resulted in new relationships between entities being created and discovered [34]. |
| User Experience | F | The authors claim that IPUMS is findable because the user interface is carefully designed to ensure that users can locate the specific data they need for their research. IPUMS staff and enthusiastic users also actively promote the data resources through workshops and conference presentations, publications, use in classrooms, blogs, and social media. They also claim that they strive to make the interfaces as easy to use as possible, enabling users to navigate large data collections [25]. |
| Costs of Data Access to User | A | The authors claim that IPUMS is accessible because data are provided free of charge and with as few restrictions as possible [25]. |
| FAIR training as you release your FAIR software/infrastructure/ tool. | F, A, I, R | The authors claim that IPUMS is FAIR because it contains an extensive program of user training and support. Each IPUMS product provides a series of brief video tutorials on the use of the data extract systems and other aspects of data use. IPUMS also conducts in-person training and workshops at a variety of conferences. An active users’ forum allows users to learn from each other, asking and answering questions about the data. Finally, a highly responsive user support team answers phone calls and emails and monitors the users’ forum [25]. |
| Microdata coding for metadata | F | IPUMS microdata products code variables across multiple censuses or surveys. By researching questionnaires, enumerator instructions, and other documentation, IPUMS staff identify questions in each census that address the same characteristics, such as marital status, educational attainment, or occupation. Researchers then work to map the possible responses from each census to a common coding scheme while retaining as much detail of the original response sets as possible. Researchers create translation tables, a key piece of operational metadata, that map each original response code to a code in the standardized structure. Data processing software utilizes these translation tables to convert the source data into IPUMS data files [25]. |
| Record linkage | **F** | IPUMS links the same individual as they appear across multiple censuses or surveys to enable researchers to study life course. These linkages are based on matching individual characteristics—name, year and place of birth, and race—on records from each census. Unique identifiers are provided for individuals and households based on technical information from the Current Population Survey to enable tracking of individuals throughout the eight months they participate in the survey [25]. |
| Interpolation | F | A major challenge in working with census data over time is that the boundaries of administrative and statistical units change from one census to the next. IPUMS addresses this challenge through two main approaches, harmonization and interpolation [25]. |
| Structuring metadata | F, I | 3 tasks were performed to assemble sets of rich contextual metadata acquired from clinical case reports: Designed a structured data template including the primary features of most case reports and curated a corpus of case report documents along with their associated metadata records; Extracted metadata from each document in the corpus using manual and automated methods; Metadata records were aggregated into a single set of documents and verified [16]. |
| Data dictionary, DOIs, GUIDs | F, A, I, R | In the context of the Biomedical Research Informatics Computing System (BRICS), GUID does not imply findability on the web and therefore cannot be considered globally unique as implied by the principles. However, the system supports findability of research participant data within a BRICS instance. Authorized researchers can use GUID to link together all submitted information for a single participant, even if data was collected at different locations and/or for different purpose(s). The DOIs are assigned to individual research studies and are findable within the established repositories, available also from open sites with core metadata. Data quality and consistency of submissions is enhanced by validation with domain specific metadata. BRICS also provides for an automated means of mapping clinical data elements to other informatic systems metadata that are available through public websites to make data interoperable and reusable [17]. |
| COVID-19 | F, A, I, R | Addresses the need for COVID-19 distributed analytics as well as on vetting and annotation of data in near-real time; As the data are de facto distributed, rich FAIR metadata is necessary to enable controlled, computational access for analysis or visualization; Addresses the need for minimal certification scheme for all components [35]. |
| Semantic Modelling | I | The FAIR principles are applied simultaneously a highly cited drug repurposing workflow. This includes FAIRification of the involved datasets, as well as applying semantic technologies to represent and store data about the detailed versions of the general protocol, of the concrete workflow instructions, and of their execution traces. A semantic model to address these specific requirements and was evaluated by answering competency questions is proposed and evaluated [33]. |
| Cloud computing | F, A, I, R | A FAIR cloud-based platform is deployed to support a growing ecosystem of collaborators and artefacts. The technology stack uses mostly open source components that take advantage of the large storage and scalable computational environment of Amazon Web Services. The platform provides a forum for users to ask questions about the data available or analysis plans and interpretation [19]. |
| Extending the FAIR data principles | F, A, I, R | Proposes that the FAIR data principles be extended to the FAIR-Health principles to accommodate the reuse of biological material and data. The proposed FAIR-Health principles include: (1) quality and traceability; (2) incentive schemes; and (3) privacy regulation compliance [42]. |
| FAIR repository/archive | F, A, I, R | To make repositories FAIR: (1) Findable: use metadata Uniform Resource Identifiers (URIs) and Unique IDs to make data findable across geographically distributed nodes. (2) Accessible: user authentication and search engine modules would make data retrievable and provide access only to authorized users. (3) Interoperable: data read and write adapters would simplify the process of data sharing and searching across heterogeneous data sources. (4) Reusable: user interface would display all pertinent dataset information so users are able to make the decision on whether to reuse these datasets in the future [22]. |
|  | F, A, I, R | The team identified the California Teachers' Study (CTS) data that the data warehouse (DW) would contain: and formatted its datasets and accompanying documentation in standard ways to populate the data tables that would provide the foundation of the DW. The team then chose a Microsoft Remote Desktop Connection (RDC) user interface operating within a secure environment. Authorized users launch RDC to access the new CTS data environment [24]. |
|  | I, R | Data Archive allows data sharing, preservation and re-use to facilitate robust integrative cross-study data analyses for prospective studies of acute child trauma and recovery [21] |
|  | F, A, I, R | Shows the need for the creation of a FAIR-compliant data catalogue of all data assets in biopharma companies. If no company-wide support for FAIR currently exists this step could initially be scoped to a specific domain quickly to demonstrate benefits bring all data together into a virtual, federated, infrastructure, so that data with the right credentials become instantaneously accessible for human and machine interactions The gains for the biopharma sector:  Accelerated innovation owing to availability of FAIR data for primary use and secondary reuse; Reducing the time from drug discovery to market value by shortening clinical trials; Developing more-segmented or -personalised medicines by exploiting FAIR real-word data to match best treatment to relevant patient cohorts; Enabling data sharing and collaborations across institutions [23]. |
| Facilitating collaboration for FAIR research | F, A, I, R | -ELIXIR actively supports the open, collaborative spirit in the international research community where scientists are offering data and results openly to create opportunities to link data and resources and help channel community efforts into long-term sustainable infrastructures.  -ELIXIR encourages researchers to deposit their scientific data in ELIXIR’s central repository and molecular databases. This aims to ensure that COVID-19 data are well annotated and accessible for reuse by the research community and society. -ELIXIR is providing interfaces into open-access workflow and analysis environments and fast-track a minimal viable product, to provide a registry to collect COVID-19 related workflows to ensure that researchers can collaborate, publish and reuse developed workflows and pipelines [47]. |
| Pseudonymization concerns | F, I | In research projects that involve the usage of data from multiple data owners, each data owner may apply different pseudonymization methods. Therefore, a description of how each data owner sanitized the data should be provided, as well as an assessment of the data quality and data linkage across multiple data sets [31]. |
