# Supplementary material for: Initiatives, Concepts, and Implementation Practices of the Findable, Accessible, Interoperable, and Reusable Data Principles in Health Data Stewardship: Scoping Review
Source: J Med Internet Res. 2023 Aug 28;25:e45013. doi: 10.2196/45013 (PMC10495848; doi:10.2196/45013)
Supplement: Multimedia Appendix 3 [file jmir_v25i1e45013_app3.docx]

|  | | | | | |
| --- | --- | --- | --- | --- | --- |
| **Project Name** | **Type (I, W, T)** | **Objective/Research Question** | **Achievement for FAIRification** | **Limitations/ Challenges facing the FAIRification process** | **Resource link** |
| From Raw Data to FAIR Data: The FAIRification Workflow for Health Research | W | To present the architecture design of an open technological solution built upon the FAIRification process for health datasets | The FAIRification process has been examined and adapted to health data requirements considering technical, ethical, and legal implications that reusing health data for biomedical research purposes may have. | The proposed FAIRification workflow and the architecture design has not been tested in real settings yet. | Not explicit [6] |
| OpenPREDICT | W | To describe the FAIRification of datasets and applying semantic technologies to represent and store data about the detailed versions of the general protocol, of the concrete workflow instructions, and of their execution traces. | An approach used to FAIRify a highly cited drug repurposing workflow was evaluted. | Reproducibility challenges The semantic modelling of the unified workflow model was challenging | <https://github.com/fair-workflows/openpredict/tree/master/data/ontology> [33] |
| Project Tycho | T (repository) | To illustrate the value of investing in a domain-specific open-data resource for accelerating science and creating new knowledge and to describe the significant update of Project Tycho into version 2.0 with improved FAIR compliance. | Project Tycho has been significantly updated into version 2.0 with new data and improved FAIR compliance, towards a FAIR compliant data repository for global population health. | Not explicit | <https://www.tycho.pitt.edu/> [15] |
| A reference set of curated biomedical data and metadata from clinical case reports | T (template) | To expand the value of CCRs as a vital biomedical knowledge resource through extensive metadata creation. and provide a manageable, structured set of metadata on clinical events and case descriptions | This dataset itself meets the FAIR Data Principles. Out of the 9 metrics used by FAIRShake (https://fairshake.cloud/), our dataset provides all 9 of the necessary values. | Not explicit | <https://www.nature.com/articles/sdata2018258/tables/2> [16] |
| Development of an informatics system for accelerating biomedical research | I | To discuss the overall system design and an architecture that supports the various BRICS instances. Also shown is the role of individual system components that enable data to be FAIR. | The functionalities developed to use the Clinical Data Elements for electronic data submission, processing, validation and storage within designated repositories have been presented. System access is highlighted for searching across research studies within a BRICS instance. | Not explicit | <https://github.com/brics-dev/brics> [17] |
| SCALEUS-FD: A FAIR Data Tool for Biomedical Applications | T (semantic web tool) | To support the difficulties of researchers on sharing their data by publishing FAIR-compliant data and metadata to facilitate interoperability and reuse | SCALEUS-FD lightens the burden of publishing FAIR-compliant data and metadata to facilitate (meta)data integration, interoperability and reuse. It follows the Semantic Web and Linked Data principles, offering a FAIR REST API for machine-to-machine operations. | Not explicit | <https://github.com/bioinformatics-ua/scaleus> [45] |
| Applying FAIRness: Redesigning a Biomedical Informatics Research Data Management Pipeline | I | To show the FAIRification of a research data management pipeline capable of creation, storage, and data processing | The methods employed yielded increased FAIRness of data integration and data storage solutions, but lack enabling more FAIR Guiding Principles regarding Data Usage. The FAIRmetrics were not applicable to our analysis of software tools. | The FAIRmetrics were not applicable to the analysis of software tools used. | Not explicit [28] |
| Development of the Prospective studies of Acute Child Trauma and Recovery (PACT/R) Data Archive | T (Data Archive) | To describe the creation of a research resource, including harmonization of key variables; describe key study- and participant-level variables; and examine retention to follow-up across studies | To enable researchers to better examine the nature and course of children’s responses to acute trauma exposure by combining data from multiple studies through enhanced data stewardship. | Not explicit | <https://www.childtraumadata.org/datasets-pactr-archive> [21] |
| FAIR data for next-generation management of multiple sclerosis | W | To describe the method to revolutionize management of MS to a personalized, individualized and precision level using FAIR data. | Transforming the current population-based management into an individualized, personalized and precision-level management is a major goal in research. Here, a method to revolutionize management of MS to a personalized, individualized and precision level via FAIR data is outlined. | Not explicit | <https://www.uhasselt.be/msdataconnect> [43] |
| The YOUth cohort study | W | To describe how a data infrastructure has been built for a large longitudinal cohort. One part focuses on the rather IT-related problems, another section focuses solely on the FAIRification tasks. | The model of data quality control and data stewardship puts a large, sensitive and complex cohort study at the forefront of FAIR data infrastructure | Not explicit | Not explicit [36] |
| Menoci: lightweight extensible web portal enhancing data management for biomedical research projects | T | To introduce the modular web portal software menoci for data collection, experiment documentation, data publication, sharing, and preservation in biomedical research projects. | This software focuses mainly on the collection and integration of data, and the comprehensive documentation and workflow support. | Not explicit | <https://menoci.io> [29] |
| Insights from adopting a data commons approach for large-scale observational cohort studies: the California Teachers Study | I (California Teachers Study Data Warehouse) | To describe the development, deployment, and features of the new FAIR cloud-based approach for storing, analyzing, and sharing CTS data in one common, secure, shared environment adopted by the CTS in 2014. | In 2013, the California Teachers Study (CTS), a multi-site prospective CEC of 133,479 women, was using the same data strategy, analysis infrastructure, and IT resources that it had used since 1995. The CTS stored its data on local network drives at CTS investigators' institutions, but this created data silos that hindered real-time collaboration. The CTS manually merged, updated, and distributed individual and summary datasets for analyses and data sharing, but this was time-consuming (2) and less efficient than other CECs that were storing data in a centralized server (6) or repository (7) environments. In 2014, the CTS adopted a new cloud-based approach for storing, analysing, and sharing CTS data in one common, secure, and shared environment. | In 2014, no template existed for a Cancer Epidemiology Cohort-specific DW. A custom data model to hedge against potential poor fit of CTS data to other observational data models based on electronic medical records was chosen. Fitting the CTS to a fully open data model, such as OHDSI, would further increase interoperability. As an early adopter among CECs, building our DW required significant up-front investment to develop a data model, configure the user interface, and convert 20 years' worth of existing CTS datasets into a single integrated DW environment. Replicating this process in another CEC would require less investment today because increasingly more options and examples exist for transitioning to fully FAIR data. The CTS DW team embraced the opportunity to learn new data science and data warehousing skills. Pivoting from every CTS investigator analysing her own copy of CTS data to investigators using shared resources requires a conceptual shift in focus from the individual investigator to the broader user community. There were differing levels of uptake across the CTS. The Windows remote desktop provides a secure environment with a familiar user interface and workbenches balance security and interoperability. | <https://www.calteachersstudy.org/> [24] |
| OpenPVSignal: Advancing Information Search, Sharing and Reuse on Pharmacovigilance Signals via FAIR Principles and Semantic Web Technologies | T | To present OpenPVSignal, a novel ontology aiming to support the semantic enrichment and rigorous communication of pharmacovigilance (PV) signal information in a systematic way, focusing on publishing signal information according to the FAIR data principles, and exploiting automatic reasoning capabilities upon the interlinked PV signal report data. | Current free-text based dissemination practices do not facilitate automated processing, linkage and reuse of the respective information, since this information is not provided in a “computable” format. The introduction of information technology (IT) tools and the use of semantically-enriched metadata can reinforce data expressiveness, exchange, linkage, and verification (through provenance information), as well as processing capabilities. The ultimate goal of OpenPVSignal is the advancement of current practices as regards the publication and further processing of PV signal information by focusing on two key goals: publishing information following the FAIR exploiting automated reasoning capabilities upon the interlinked PV signal report data. | Not explicit | <https://inab-certh.github.io/OpenPVSignal/> [44] |
| Interoperable and accessible census and survey data from IPUMS | I | This work describes the adherence of IPUMS and related products to the FAIR principles. Although the first release of IPUMS data in 1993 predates the FAIR guiding principles, the IPUMS philosophy has always been consistent with the FAIR principles | Not explicit | Not explicit | <https://www.ipums.org/> [25] |
| The OSSE Registry | T | To describe the first steps towards the architecture extension and implementation of the FAIR Data Principles in OSSE via an FDP. The focus is to build a first prototype. | The OSSE does not provide a limited interface to communicate data with registries of other software solutions. An open approach, with standardized vocabulary and ontologies is desirable in order to facilitate a simple connection. The FAIR Principles have been implemented in OSSE for this purpose and the architecture has been extended. The main reason to adopt FAIR in OSSE is to provide a standardized interface to communicate with registries of other software solutions and registries. The focus is to build a first prototype. | To meet the changing requirements in the field of digital health care, the European Commission proposed a comprehensive reform of data protection rules in the EU. The novel regulation will apply from 25 May 2018. Time will show if registries can remain FAIR and data privacy compliant at the same time. | <https://www.toolpool-gesundheitsforschung.de/produkte/osse> [32] |
| The French National Registry of patients with Facioscapulohumeral muscular dystrophy | T | To report the creation of the French national FSHD registry and highlight its original design allowing a strong involvement of both patients and physicians, and its evolution since 2013 | Not explicit | Not explicit | [www.fshd.fr](http://www.fshd.fr) [40] |
| The radiation oncology ontology (ROO) | T | Machine learning algorithms could be trained over clinical “big data” to build prediction models for personalized therapy. To reach this goal, a scalable “big data” architecture for the medical domain becomes essential, based on data standardization to transform clinical data into FAIR data. In this work ontologies and semantic web technologies are used to train machine learning algorithms. | The ROO allows transforming unstructured clinical data to become: Findable (F): each data entity and their properties (F2), translated into universally concept via the ROO will have a globally unique identifier (F1) and will be indexed on the Web (F3). Metadata will include specification of the data identifier (F4) Accessible (A): data will be retrievable by means of RDF triples and queryable using a universal language (A1). A permanent de-centralized storage point will be permanently available (A2), even when the original database could not be anymore. Interoperable (I): data are represented by universally adopted RDF language (I1). Queries rely on concept from imported ontologies/vocabularies that follow FAIR principles (I2). Reusable (R): several attributes specific data properties and the relations between different concepts via ROO predicates (R1). | The ROO is yet to be tested also on larger databases, other diseases and routine clinical data to check if all the main information is covered. This work lacks of the system evaluation. Further investigations on evaluating the system performance need to be considered such as comparing the query time between SPARQL and traditional databases. | <https://bioportal.bioontology.org/ontologies/ROO> [37] |
| Connecting data, tools and people across Europe: ELIXIR’s response to the COVID-19 pandemic | I | To describe the response and actions taken by national Nodes—and note that this strategy directly answers to priority action 8 (“Access to research infrastructures”) and action 9 (“Research data sharing platform”) in the EU action plan for COVID-19. | The research response to the COVID-19 has led to the development of new therapeutics, drug repurposing, clinical studies to e.g. provide data on epidemiological characteristics, host susceptibility and host immune responses, risk factors for severe disease and routes of transmission. Collectively, the launched national and European projects present a major data generation effort. Ensuring that data are available for access across teams and countries is imperative for the rapid response to the COVID-19 outbreak. ELIXIR will also work with communities to remove obstacles to efficient sharing, for instance the technical interoperability of datasets. A key point for reuse is the quality of the metadata annotation. Many ELIXIR nodes provide data management support to projects launched nationally and at the EU level such that data are published for broad access and reuse. A particular challenge is data from healthcare providers (“medical data”) and other actors in the healthcare systems (“real-world data”). Discovering, accessing and linking such data is imperative in the response to COVID-19—and extraordinarily challenging, as data from the healthcare systems are encoded with many different standards and governance models. National infrastructures will—as part of the COVID-19 response—engage with the many European stakeholders and ELIXIR will seek to further the development and application of normalisation and interoperability of medical and real-world data by seeking collaborations with projects such as IMI EDHEN and FAIR4Health. | Data from healthcare providers (“medical data”) and other actors in the healthcare systems (“real-world data”). Discovering, accessing and linking such data is imperative in the response to COVID-19—and extraordinarily challenging, as data from the healthcare systems are encoded with many different standards and governance models. National infrastructures will—as part of the COVID-19 response—engage with the many European stakeholders and ELIXIR will seek to further the development and application of normalisation and interoperability of medical and real-world data | [elixir-europe.org/services/covid-19-resources](https://elixir-europe.org/services/covid-19-resources). [47] |
| ClinEpiDB: an open-access clinical epidemiology database resource encouraging online exploration of complex studies | I | To present an intuitive point-and-click website that allows users to visualize and subset data directly in the ClinEpiDB browser and immediately explore potential associations. Supporting study documentation aids contextualization, and data can be downloaded for advanced analyses. By facilitating access and interrogation of high-quality, large-scale data sets, ClinEpiDB aims to spur collaboration and discovery that improves global health. | 1. Researchers supply flat data files along with data dictionaries, data collection forms, and protocols to help contextualize the data. Variables within the data set may contain categorical, continuous, discrete, or free text data. 2. Once the data are received, a series of files are constructed according to a standard operating procedure to process the variables, map them to ontology terms, and map coded categorical values to the descriptive terms. 3. Once the data, ontology, and value mapping files are prepared, the data undergo processing to obfuscate dates to protect participant confidentiality. All dates for a given participant are consistently shifted forward or backward by 0–7 days according to a random number algorithm.  4. All data are then transformed into an ISA-based format (Sansone et al., 2012) and loaded into a relational database based on the Genomics Unified Schema, version 4 (GUS4) ( Davidson et al., 2001) running in an Oracle database management system (DBMS). Build database servers are located at the University of Pennsylvania and production instances are mirrored at the Universities of Pennsylvania and Georgia for redundancy purposes and to ensure uptime. All servers are housed in FISMA-compliant computational facilities, and industry standard backups of all data are performed. 5. Searches for each study are made available to users in an intuitive user interface (the “Search Wizard”), driven by a series of SQL queries against the GUS4 database. 6. Exploration applications for additional data visualization are created with Shiny, an open-source R package for building interactive web applications 7. The applications are hosted on the website via the Shiny Server Open Source software. SQL queries against the Oracle database identify all variables in the study and their format, which informs which variables appear as options to plot, how to build a custom dichotomous variable, and how the data are plotted within the applications. 8. Studies are reviewed by ClinEpiDB staff for quality control and made accessible to primary data providers using a protected internal website to ensure data accuracy and query functionality. Data are only scheduled for public release following data provider approval. Updates to the database are released every two months and can include new studies, features, and/or software updates. | **Not explicit** | https://eupathdb.org/eupathdb/wdkCustomization/jsp/questions/XmlQuestions.Infrastructure.jsp Source code available from: <https://github.com/VEuPathDB> [46] |
| American Heart Association Precision Medicine Platform | T | To introduce the FAIR-based American Heart Association Precision Medicine Platform. The platform's goals are to democratize data, make it easy to search across orthogonal data sets, provide a secure workspace to leverage the power of cloud computing, and provide a forum for users to share insights. | When accessing large public data sets today, researchers have to find, access, download, and interpret each data set individually. Researchers must untangle and interpret the data and then expend resources to house the data. In addition, the lack of harmonization across multiple data sets obviates the ability of researchers to combine data sources and to confirm or generate cogent findings. | 1. Ensuring platform scalability, security, privacy, and ease of use.  2. The community’s perceived lack of information safety in cloud computing.  3. Balancing the protection of intellectual property with enabling collaboration. To overcome this, the community search portal allows only summary-level views of results; detailed views are available only to those who own or have been granted access to data in private workspaces. | [https://precision.heart.org](https://precision.heart.org/) [19] |
