# Supplementary material for: Initiatives, Concepts, and Implementation Practices of the Findable, Accessible, Interoperable, and Reusable Data Principles in Health Data Stewardship: Scoping Review
Source: J Med Internet Res. 2023 Aug 28;25:e45013. doi: 10.2196/45013 (PMC10495848; doi:10.2196/45013)
Supplement: Multimedia Appendix 4 [file jmir_v25i1e45013_app4.docx]

| **FAIR axis** | **Sub categories** | Challenges description | **Used/suggested mitigation approach** |
| --- | --- | --- | --- |
| **Findability** | **Identifies issues** | Many data repository software systems or content management  systems assign (locally) unique numbers by themselves, which could be made globally unique by a suitable prefix.  The required "eternal persistence” for locally assigned unique numbers cannot be guaranteed with regard to updates or system changes. | FHIR uses globally unique identifiers and can assign other identifiers [6,30]. |
|  |  | Despite the importance of HL7 Object Identifier (OID), Digital Object Identifiers (DOI), and Uniform Resource Identifiers (URI) in the medical field, there is no agreement on desirable characteristics of human readable designation as part of identifiers |  |
|  |  | The level of granularity at which data is to be assigned its identifiers is not easy to determine due to the constant addition and update of medical databases |  |
|  | **Community oriented metadata vocabularies** | Navigation within a repository is hampered due to a lack of community-accepted metadata vocabularies for describing datasets for many areas of biomedical research. | The semantic model for the dataset should incorporate vocabularies, terminology systems, coding standards such ICD,SNOMED CT and LOINC [6]. |
|  |  | lack of a unifying data catalog specification for available health data describing the content of data collections and the location |  |
|  | **Content driven searching tools** | Lack of data dictionary and tools to support users for content driven searches for data left users with little insight into the content of the data unless they download the full datasets or follow links to the original source of the data. (Bhatia, Tanch et al. 2020) DOI https://doi.org/  10.1055/s-0040-1712510. | Improving Accessibility and Interoperability by  Including Data Dictionaries in Metadata [27]. |
|  | Storage and archival requirements | The diverse format, size, continuity and duration of data collection require a high level of reliable and durable storage infrastructure.    Data collection across different legal entities imposes additional regulatory burdens on the data collection, such as data processing agreements  (Zondergeld, Scholten et al. 2020) 10.1016/j.dcn.2020.100834 | Setting a dedicated storage solution for each tabular data (e.g. questionnaire responses), non-tabular data (like EEG or MRI measurements), and biological material [36] |
|  | Performance | Using cloud computing for some types of data, e.g. genomic data, requires lengthy data transfers for uploading data to the cloud serve. | A portion of genomics data today exist in cloud-based environments, making this challenge feasible [19] |
| **Accessibility** | **Data Access protocol** | The accessibility of the YOUth data is necessarily restricted.  Handling a data request in a multi-step procedure and involving multiple actors requires a system to ease access. | Development of an automated data request processing system to streamline the handling of data requests and increase the ease of accessibility of the YOUth data. It allows researchers to submit data requests online and facilitates the actions requested from the people involved in the evaluation and transfer procedures [36]. |
|  | **Cultural issues** | Permanent availability of data, even when the data are no longer available, is technically relatively easy to achieve but harder to enforce culturally as it is not yet fully endorsed by some researchers. | The culture of the workforce needs to evolve from ‘it’s my lab and my data’; rather ‘it’s the company’s data and its FAIR’ [30]. |
|  |  | A cultural challenge is needed to move an organisation from a protective and siloed data mentality to a mindset of data sharing with all relevant stakeholders. | Implementation of FAIR requires an incentivised, cultural change, a combination of top-down commitment and investment from senior management, along with a bottom-up approach from scientists and managers [23] |
|  |  | Cohort studies stored and archival practices on local network drives at the various investigators' institutions hinder real-time collaboration. | Replacing the data management environment/platform with infrastructure and workflows can increase access to cohort data, removes many barriers to use, and accommodates data storage, cleaning, updating, analysing, and sharing [24].  Pivoting from every study investigator analysing the copy if their data to all investigators using shared resources requires a conceptual shift in focus from the individual investigator to the broader user community [24]. |
|  | **Data politics** | Access to the required data from the Real-World Observations (RWO) on the current pandemic is severely limited due to the politicisation of the data. | To seek collaborations with institutions that work on established knowledge (EK) bases and genuine partnerships around the globe.  Embrace the use of the COVID-19 situation as a rapid application of the core infrastructure of the European Open Science Cloud.  Need to facilitate and further enhance infrastructure and methods for ‘distributed deep learning’ [35]. |
|  | **GDPR restriction** | GDPR comprehensive regulation sets several conditions and restrictions for data collection, including detailed consents research by stating the objective, the persons accessing the data and the circumstances of the data processing, which can prevent subsequent data sharing. | Create and use further harmonised metadata vocabularies on topics such as the legal basis for data collection or the different variants of Informed Consent [30,35]. |
|  |  | Data collection or record linkage is more difficult as identifying data must not be stored together with other research data. | If data sets are not amenable to sharing (privacy, scope), privacy-preserving data analysis techniques could be an option [30,35] |
|  |  | Sensitive personal data cannot be made publicly available, and therefore need to invest in long-term management of the data infrastructure and procedures regulating the sharing of data | More (long term) funding for projects that produce large amounts of high-quality, FAIR data that is securely stored and managed is therefore crucial if we want to make science more sustainable [36]. |
|  |  | Lack of awareness of legal frameworks on research data access and the contact person for a given data record is a major hurdle for both the data holder and the interested party. | The establishment of a Data Access Board for checking and approving data use proposals according to a usage concept is strongly recommended [30]. |
|  | **Privacy breach and safety concerns** | Concerns of data owners about possible breaches of data privacy during data sharing. E.g. Perceived lack of information safety in cloud computing by the professional communities. | Configure the cloud computing infrastructure, computation, and software platform with diverse security, confidentiality, and authentication settings to adhere to widely adopted national and international standards and regulations  Verify the platform by A third-party assessor for the suitability of the environment to be compatible national law for analysing biomedical data in a cloud-based environment [19,30] |
|  | **Balancing security with access** | Safekeeping of highly privacy-sensitive data on the one hand and facilitating the scientific community’s access to these rich and unique data on the other hand. | The community search portal allows only summary-level views of the results [36]. |
|  | **Personal and Economic aspects** | Data access of claims data refers to the “accessible” dimension of the FAIR guiding principles. Extrinsic factors such as human and economic factors influence the accessibility of claims data. | Not explicit [39]. |
|  | **Reproducibility challenges** | FAIR principles apply to various domains of science but are not specific enough to deal with the major challenges of medical research, such as reproducibility and privacy protection | Propose an extension of the FAIR Principles to include the following components: quality aspect, incentives to stimulate effective enrichment of data sets and biological material collections and privacy-respecting approaches for working with the human material and data. [42]. |
| **Interoperability** | **Adherence to standard vs freedom of science** | In biomedical research, there are countless standards, conventions, and best practices, but researchers use the freedom of science to act on their own ideas in many cases.  Lack of domain specific template forced the use of a custom model, which limited the interoperability. | Since the requirements for technical and semantic interoperability are usually higher, operators should support data owners in converting the data into a generally usable format for knowledge representation [30]. |
|  | **Standards variation** | Data from the healthcare systems are encoded with many different standards and governance models. | ELIXIR will seek to further the development and application of normalisation and interoperability of medical and real-world data [47]. |
|  |  | Successful deployment of FAIR will require a standardised information architecture | Biopharma should reach a robust consensus on the ontologies they use to capture specific types of data [23].  The OSSE Metadata repository offers the possibility to uniformly describe and define data elements and is used as an additional tool for creating semantic interoperability in a FAIR infrastructure [32]. |
|  | **Semantic modelling development** | The challenge of creating a new ontology from scratch rather than creating a unified model based on the existing ontologies.  The semantic modelling of the unified workflow model was challenging, an extensive task of reusing existing semantic approach workflow implementation consistency issues such as missing disjoints and licensing elements. | The semantic models were reused which improved semantic interoperability as opposed to creating a new ontology which may impede interoperability if it is not accompanied with alignments to the existing semantic models [6].  DataCite standard from European Clinical Research Information Network (ECRIN) group can serve as a starting point for those public health care providers and health researchers wishing to FAIRify their datasets for research purposes [33]. |
|  | **Security and interoperability** | The is a need to balance security and interoperability. | Describes a Windows remote desktop provides a secure environment with a familiar user interface, but this approach also puts limits on external connections [24]. |
|  | **Technology** | There is limited understanding and availability of technology and standards to support FAIR implementation in biopharmaceutical industry. | Biopharmaceutical companies need to work closely with the technology supply companies to increase support for the FAIR principles through pre-competitive activities such as industry-wide training, development and education programmes [23]. |
|  | **Terminology/semantic difference** | The use of different terminology for similar conceptualizations is challenging. E.g. the definitions of terms like plan, process, protocol, procedure, workflow, plan specification and standard operating procedure seem to be the same (or quite overlapping), their meanings become notoriously ambiguous across varied communities. | How to grasp these semantic differences is a crucial question that needs further exploration [33]. |
| **Reusability** | **Provenance** | Provenance is a broad topic, and the demarcation to data acquisition and processing circumstances is not sharp. | The provision of simple web-based visual analytics tools that give potential prospects an overview of the depth of available data can increase the reusability [30] |
|  | **Data quality** | Data quality is an important concern for primary data and when relying on external data. | Ensuring data quality through technical validations and manual maintenance should be explicitly mentioned. [30,36] |
|  | **Incentive** | The effort and benefits of FAIR review of data are generally unevenly distributed and are at the expense of the data owners. Implementation of FAIR requires an incentivised, cultural change driven by bottom-up and top-down approaches | We need to incentivise the data owners  To achieve such a cultural change, organisations should provide incentives to share data, support activities to enhance awareness, such as training, and promote methods to assess and support change management [30]. |
|  | **Cost/ROI** | High costs are encountered in the key business categories of people, process, technology and data.  Require a significant up-front investment to develop a data model, configure the user interface, and also convert decade years’ worth of existing datasets into a single integrated data warehouse (DW) environment | Executive management will need to be convinced that FAIR implementation will generate a long-term ROI as well as being a high-priority, urgent endeavour [23].  A grant to facilitate the democratization of data through a new and more efficient Data warehouse infrastructure and replicating this process in another similar cohort would now require less investment [24]. |
|  | **Human and infrastructure aspect** | Production of high-quality, FAIR data while safeguarding the privacy of participants requires an extensive data infrastructure, set up by collaborative efforts of stakeholders in the data life cycle (e.g. Researchers, data managers, IT departments, and the University Library) | Coordinate the partner organizations with a clear ambition to support proper data management and accessibility through institutional support which include a dedicated research IT division and high-quality data managers  Crucial to the long-term success of FAIR implementation is the involvement of all data stakeholders across all functions and along the complete data lifecycle [36]. |
