# Supplementary material for: Initiatives, Concepts, and Implementation Practices of the Findable, Accessible, Interoperable, and Reusable Data Principles in Health Data Stewardship: Scoping Review
Source: J Med Internet Res. 2023 Aug 28;25:e45013. doi: 10.2196/45013 (PMC10495848; doi:10.2196/45013)
Supplement: Multimedia Appendix 5 [file jmir_v25i1e45013_app5.docx]

| **Network** | **Community/ Funding** | **Focus: What has been done?** | **Goals: What does the network want to achieve in future?** | **Resource link** |
| --- | --- | --- | --- | --- |
| Project Tycho | National Institute of General Medical Sciences Models of Infectious Disease Agent Study, the NIH Big Data to Knowledge program, and the Bill and Melinda Gates Foundation. | Project Tycho version 1 data has been used to create new knowledge and technology that facilitates research in global health. The FAIRification of the version 1 was the basis on which version 2 was developed. | Not Explicit | <https://www.tycho.pitt.edu/> [15] |
| W3C Semantic Web for Health Care and Life Sciences Interest Group (HCLS) | NIAID through funds provided by the trans-NIH Big Data to Knowledge (BD2K) initiative; Open PHACTS, European Union’s Seventh Framework Programme, EFPIA companies; European Commission; US National Institutes of Health grant; Swiss Federal Government; BBSRC Institute Strategic Programme; Integrated Database Project (Ministry of Education, Culture, Sports Science and Technology, Japan), the National Bioscience Database Center (NBDC - Japan), and the Database Center for Life Sciences (DBCLS - Japan). | A multi-stakeholder effort was conducted to produce a specification for the description of datasets that meets key functional requirements, uses existing vocabularies and is expressed using the RDF. This work describes elements of data description including provenance and versioning. | To add new use cases and document improvements made to the existing community profile. | <http://www.w3.org/blog/hcls/> [18] |
| Virus Outbreak Data Network (VODAN) Implementation Network (IN);  Committee on Data (CODATA), GO FAIR | GO FAIR International Science Council | CODATA aims to improve the FAIRness research data. They also established a working group to support VODAN IN which continues to increase the number of partners collaborating in the IN. This fulfils the need for community-based networking. | To provide a platform for FAIR data exchange during the COVID pandemic and a reference point for data stewardship in future pandemics; Research into the application of data to real world issues; Promote application of principles, policies and practices that enable Open Data and Open Science; Advance data science by improving data skills and the functions of national science systems. | [https://codata.org/; https://council.science/about-us/) https://www.go-fair.org/implementation-networks/overview/vodan](https://codata.org/;%20https://council.science/about-us/)https://www.go-fair.org/implementation-networks/overview/vodan) [35] |
| The American Heart Association | Powered by Amazon Web Services | The platform provides a transparent and explicit harmonization access to both harmonized and raw data. | Future plans include involving the community participating for better data diversification. | <https://precision.heart.org>; <https://professional.heart.org/en/research-programs/aha-research-policies-and-awardee-hub/open-science-policy-statements-for-aha-funded-research> [19] |
| DATS, bioCADDIE | National Institutes of Health (NIH)’s Big Data to Knowledge (BD2K) initiative | Designed and implemented the Data Tag Suite (DATS) model to support the DataMed data discovery index | To develop a tool that enables the discovery of available biomedical datasets that are spread across different databases and on the cloud | <https://biocaddie.org/> [20] |
| BBMRI-ERIC, European Research Infrastructure Consortium (ERIC) | Initially by EU since 2013 it is a legal entity | Extended the FAIR principles to FAIR health principles in biological material management through providing comprehensive provenance information for the complete chain from a donor to biological material to data, as well as incentives for enriching existing resources and reusing them | Not Explicit | [www.bbmri-eric.eu/](http://www.bbmri-eric.eu/) [42] |
| FAIRSharing | EU European Open Science Cloud - EOSC, EU H2020, European Research Council - ERC, UK Open Research Data Task Force | Has guided consumers to discover, select and use these resources with confidence, and producers to make their resources more discoverable, more widely adopted and cited. | To grow the number of users, adopters, collaborators and activities, all working to enable the FAIR Principles and to make Standards, Knowledge bases, Repositories and Data Policies FAIR. | <https://fairsharing.org/> [29] |
| ELIXIR | Intergovernmental organisation that brings together life science resources from across Europe. | ELIXIR works with communities to remove obstacles to efficient collaborative COVID-19 data sharing and metadata annotation. Also provides data management support to projects launched nationally and at the EU level such that data are published for broad access and reuse. ELIXIR provides resources for the Global COVID19 BioHackathon that create opportunities to link data and resources and help channel community efforts into long-term sustainable infrastructure. | ELIXIR plans to collaborate with appropriate stakeholders to connect researcher-driven initiatives and develop open reproducible tools and workflows for COVID research. | <https://elixir-europe.org/>; <https://github.com/virtual-biohackathons/covid-19-bh20/wiki> [47] |
| Dutch Research Council; Data Archiving and Network Services organisation (DDI Alliance) and UK Data Service | Gravitation programme of the Dutch Ministry of Education, Culture, and Science and the Netherlands Organization for Scientific Research | Development of a high-quality research data infrastructure for sensitive cohort data. | Not Explicit | <https://www.nwo.nl/en/researchprogrammes/gravitation> [35] |
| SDSC Sherlock San Diego Supercomputer Center | National Cancer Institute | Deployment of a new infrastructure based on a data warehouse to integrate and manage data and a secure and shared workspace with documentation, software, and analytic tools that facilitate collaboration and accelerate analyses. | To expand the geospatial, comorbidities, and biospecimen tools for query and analyses. Automate the process of exploring, requesting, and revising project-specific data. Development of an API to enable data collection and sharing. | <https://sherlock.sdsc.edu/> [24] |
| Collaborative Research Centers at the Göttingen Campus; DFG; FAIRSharing | Not Explicit | Development of a modular web portal software for data collection, experiment documentation, data publication, sharing, and preservation in biomedical research projects. | Not Explicit | <https://menoci.io/> [29] |
