# Supplementary material for: Initiatives, Concepts, and Implementation Practices of the Findable, Accessible, Interoperable, and Reusable Data Principles in Health Data Stewardship: Scoping Review
Source: J Med Internet Res. 2023 Aug 28;25:e45013. doi: 10.2196/45013 (PMC10495848; doi:10.2196/45013)
Supplement: Multimedia Appendix 6 [file jmir_v25i1e45013_app6.docx]

| Reported Outcome category | Description of actual outcome | Principles addressed (FAIR) | Future work/anticipation |
| --- | --- | --- | --- |
| Return in investment, new findings/treatments | Integrated linked data from several databases to support the identification and validation of drug targets, semantic integration across several public databases to support drug discovery, establishment of mechanism-based disease taxonomies for neurodegenerative diseases which are linked to clinical data for drug discovery. | F, A, R | Expected impact: (i) better findability for existing data; (ii) quicker access to the data at scale; and (iii) ready selection of standardised and high-quality data for analytics such as machine learning. (iv) increase the return of investment metrics based on lower costs (v) faster timelines to discover and develop new therapeutic treatments (vi) increase in productivity of the R&D pipeline [23] |
| Data sharing | FAIR Data Point; the OSSE Metadata repository (MDR) offers a possibility to uniformly describe and define data elements. The MDR can thus be used as an additional tool for creating semantic interoperability in a FAIR infrastructure. | I | NA [32] |
| Data sharing and return on investment | Semantic enrichment of the rich, licensed metadata has increased the internal data enabling the data managers to respond to a large variety of data requests. An automated data request processing system for access regulation has been developed. | F, A, I, R | Data reuse and sharing may cut the cost for data collection. Subsidising the implementation of safe, FAIR and high-quality datasets is therefore in the end a scientifically, and potentially also economically, sound investment [36]. |
| Reuse | The project created a sustainable framework for standard variable names, metadata, and harmonization algorithms. It has enabled new analyses of these data by investigators around the world. | F, A, R | Other approaches to harmonization of these data, such as rescaling to achieve common item response scales across different measures or using latent variable approaches to build measurement models may be applied to fit the research questions being addressed [21]. |
| Data sharing, reuse | This tool lightened the burden of publishing and sharing FAIR-compliant (meta)data to facilitate interoperability and reuse by use of semantics and linked data. | F, A, I, R | To improve the visualization interfaces of semantic data and add support for federated queries using external datasets [45] |
| Data sharing and reuse, publication | ClinEpiDB: an open-access clinical epidemiology database resource encouraging online exploration of complex studies | I | Users can expect to see the release of additional studies focusing more tropical diseases. Additional long-term development plans include strengthening and expanding data visualization and exploration tools [46]. |
| Data reuse | The advantages this tool are: (1) Facilitates the reusability  (2) Facilitates the automation of manually conducted work. (3) Interlinks the respective information with other data sources and by applying semantic reasoning. (4) Uses Linked Data and OpenPVSignal to facilitate detection of duplicate information and facilitate better processing. | F, A, I, R | (a) the development of a tool to facilitate the automatic population of OpenPVSignal with the content of already released PV signal reports by applying Natural Language Processing techniques, in order to construct the respective knowledge graph (b) the development of a user-friendly tool to create, publish, browse and query OpenPVSignal instances, appropriate for use by PV signal monitoring organizations and drug regulatory authorities (c) the development of a knowledge-based, computational framework for assessing candidate PV signals by use of the semantic capabilities contained in OpenPVSignal.  [44] |
| ROI, Publlication | The ROI will be further prevention of duplication and the acceleration of new science and discovery in global health through improved FAIR compliance of the Project Tycho data repository. Broader and more efficient reuse of disease surveillance data for research as a result of improved FAIR representation of (meta)data | F, A, I, R | A set of standard vocabularies and ontologies were used to represent disease surveillance data, but many additional standards exist and may be applicable. It would be relevant for the global health community to develop a collection of preferred, existing standards and ontologies that can be used by most stakeholders. In addition, new standards and ontologies could be created for data attributes that cannot easily be represented with current standards [15]. |
| New findings/treatments,an educational resource for writing better case reports | FAIRifying clinical case reports facilitates their discovery and study for research of treatment. This work resulted in a standardized metadata template and metrics and a test dataset consisting of 3,100 CCRs spanning 16 disease categories. The added rich metadata helps researchers develop novel approaches to medical science and patient care.  - Educational resource: Implementation of the standardized metadata template, indexing and categorization to enrich these documents can expand the audience and application of case reports. | F | NA [16] |
| Data Sharing | The OSSE-MDR offers a possibility to uniformly describe and define data elements. The MDR can thus be used as an additional tool for creating semantic interoperability in a FAIR infrastructure. | I | It is planned to implement a decentralized registry infrastructure with OSSE registries and the FAIR approach in the L-ACMAG (Longitudinal Study Registry of Aortic, Myocardial, Arterial and Genetics in aortic diseases). L-ACMAG aims to implement a data privacy compliant research collaboration among fifteen GermanReference Centres to improve the diagnosis and treatment of patients with rare genetic vascular diseases [32]. |
| Data sharing and reuse | This project combined data from many smaller research studies to enable new analyses and insights so that prospective child trauma data can be shareable, preserveable, and reusable to advance more robust science and support evidence-based approaches to treat acute paediatric trauma. | R | NA [21] |
| Data reuse | The project has created a sustainable framework for standard variable names, metadata, and harmonization algorithms, and has already enabled new analyses of these data by investigators around the world. | R | NA [21] |
| Publication | the PACT/R Archive has fulfilled data requests from investigators in North America, Europe, and Australia, resulting in two peer-reviewed publications (Kassam-Adams et al., 2012; Lenferink et al., 2020), others in preparation or under review, and two completed doctoral theses (Vibhakar, 2018; Walker, 2018). These projects demonstrate the potential impact of this growing set of accessible, harmonized child trauma data to enable novel analyses utilizing large sample sizes. | R | NA [21] |
| Data reuse | This paper presents the value of sharing historical epidemiological data for creating new knowledge and technology with the example of Project Tycho v1 and improvements made for Project Tycho v2. | I, R | The improved FAIR representation of Project Tycho (meta)data will lead to a broader and more efficient reuse of disease surveillance data for research and technology development [15]. |
| Publication | 150 published works that cited the Project Tycho release paper, 47 published by authors from one of the 100 institutions most commonly listed as affiliation by registered Project Tycho users. | R | NA [15] |
| Data sharing | CTS DW and shared workspace is consistent with the NCI CRDC Framework and provides comprehensive yet flexible infrastructure for storing, managing, analyzing, and sharing CEC data. The new CTS data infrastructure provides a foundation for continuous and ongoing development and expansion. It has unique features but shares enough common characteristics to facilitate widespread data harmonization, pooling, and sharing. The CTS now offers all users a shared and secure workspace with common data, documentation, software, and analytic tools to facilitate use and data sharing. | A, R | To expand the geospatial, comorbidities, and biospecimen tools for query and analyses. The process of exploring, requesting, and revising project-specific data can be further automated. Development of an API can facilitate data collection and sharing. Moving beyond data storage and having more high-value CEC data available could generate even greater return on the significant investments made and resources that exist in CEC [24]. |
| Publication | The research produced by these users is published in numerous articles, books, and papers, including almost 2,000 results in Google Scholar in 2015. The pace of IPUMS-based publications continues to accelerate. | I | Application programming interfaces will be launched to further extend the interoperability of IPUMS data.  Additional funding sources will be sought as an organizational structure that allows key stakeholders, including data producers and data users, to contribute to IPUMS governance will be established [25]. |
| Publication | Expands the notions of FAIR and FAIRification from the relatively static artifacts of datasets to the dynamic processes of workflows. Published the workflow representation, data and meta-data in a triple store which was used as FAIR data point. | F, A, I, R | Improve the modelling of manual steps by studying and possibly incorporating predicates from the SMART protocol’s ontology. Characterize the abstraction levels of workflows based on multi-level process modelling approaches [33]. |
| Data sharing and return on investment | "Expected benefits of DiiS-like data integration are": Simplifying exchange of electronic information; Decreasing the cost and complexity of interfacing between different systems; Improving data interoperability; Facilitating data discovery and supporting reproducibility in research | F, I | NA [22] |
| Data sharing | Simplifying exchange of electronic information. | I | NA [43] |
| Return on investment and data reuse | Decreasing the cost and complexity of interfacing between different systems; Improved data integration solutions: intergration of non-proprietary output data formats preferred by the Data Archiving and Network Services organization (DANS) of the Royal Netherlands Academy of Arts and Sciences (DDI Alliance, 2018) and by the UK Data Service (UK Data Service, n.d.); Intergrated machine-readable metadata are key to increasing the findability and the assessment of the reusability of data. | R | NA [36] |
| Data reuse | This work shows that the efficiency of a data access policy should be quantitatively and qualitatively monitored to understand the potential leverages of data reuse. | A, R | NA [41] |
| Data sharing, Reuse, Publication | The tool facilitates standardization and high-quality documentation of experiments and derived data along the scientific process as well as an easy-to-use researcher interface to cross-link and publish relevant datasets unambiguously with the manuscripts in scientific journals. Also enables collection of experimental data in a harmonized rich metadata schema and data sharing with public repositories, Uses the Drupal framework to regulate data access. | F, A, I, R | FUTURE: (1) expansion of JSON-LD API for semantic data retrieval; (2) quality assurance and integration with third party tools and systems [29]; |
| NA | NA | NA | Efforts and costs are currently too high for many common data collection projects and it helps if incentives are put in place to motivate stakeholders to take on the challenge of FAIRification. Furthermore, there should be more standardization on processes for data access and data extraction [30]. |
| New findings/treatments | TB DEPOT was developed as a unique tool to utilize the wealth of metadata contained within the TB Portals, readily displaying and analyzing it to facilitate hypothesis generation and testing, aimed at improving MDR-TB patient diagnostics and outcomes. | F | NA [26] |
| NA | NA | NA | Future developments to link rare disease resources should consider using multiple ontologies, dictionaries and terminology systems [40]. |
| NA | NA | NA | To improve the data quality of the Transp-db, it is recommended that all professional identifiers should be validated beforehand. In the objective of European collaborative initiatives, the deployment and mandatory use of a shared European identifier for healthcare professionals would guarantee a better transparency  The assessment and improvement of the quality of open data, and particularly regarding conflicts of interest is crucially, especially considering the movement toward open and shared data and the construction of European studies Further investigations on evaluating the system performance need to be considered such as comparing the query time between SPARQL and traditional databases [41]. |
| Data sharing | clinical data following FAIR principles using the combination of ontologies and Semantic Web technologies. | I | NA [31] |
| Data sharing | FAIRified clinical data  Better data integration and uses Semantic ontologies to efficiently and easily query data from different sources. | I | NA [49] |
| NA | NA | NA | ELIXIR will collaborate with appropriate stakeholders to connect researcher-driven initiatives, and develop open reproducible tools and workflows for COVID research [47]. |
| NA | NA | NA | EDCat, will be developed and evaluated further to include datasets from a wider variety of sources [27]. |
